# Evolution of outcome and complications in TAVR: a meta-analysis of observational and randomized studies

**DOI:** 10.1038/s41598-020-72453-1

**Published:** 2020-09-23

**Authors:** Max-Paul Winter, Philipp Bartko, Felix Hofer, Martin Zbiral, Achim Burger, Bahil Ghanim, Johannes Kastner, Irene M. Lang, Julia Mascherbauer, Christian Hengstenberg, Georg Goliasch

**Affiliations:** 1 Department of Internal Medicine II, Medical University of Vienna, Waehringer Guertel 18-20, 1090 Vienna, Austria; 2 Department of General and Thoracic Surgery, University Hospital Krems, Karl Landsteiner University of Health Sciences, Krems an der Donau, Austria

**Keywords:** Outcomes research, Interventional cardiology

## Abstract

Aim of the present analysis was to collect and pool all available data currently in the literature regarding outcomes and complications of all approved TAVR prosthesis and to assess the transition from first to next generation TAVR devices by directly comparing both in regard of procedure related complications. Transcatheter aortic valve replacement is a well established treatment modality in patients with severe aortic stenosis deemed to be inoperable or at unacceptable risk for open heart surgery. First generation prostheses were associated with a high rate of peri-procedural complications like paravalvular regurgitation, valve malpositioning, vascular complications and conduction disorders. Refinement of the available devices incorporate features to address the limitations of the first-generation devices. A PRISMA checklist-guided systematic review and meta-analysis of prospective observational studies, national and device specific registries or randomized clinical trials was conducted. Studies were identified by searching PUBMED, SCOPUS, Cochrane Central Register of Controlled Trials and LILACs from January 2000 to October 2017. We extracted and pooled data on both mortality and complications from 273 studies for twelve different valves prostheses in a total of 68,193 patients. In second generation prostheses as compared to first generation devices, we observed a significant decrease in mortality (1.47 ± 1.73% vs. 5.41 ± 4.35%; p < 0.001), paravalvular regurgitation (1.75 ± 2.43vs. 12.39 ± 9.38, p < 0.001) and MACE. TAVR with contemporary next generation devices has led to an impressive improvement in TAVR safety driven by refined case selection, improved procedural techniques and increased site experience.

## Introduction

Transcatheter aortic valve replacement (TAVR) has become a valuable therapeutic standard for patients with symptomatic severe aortic stenosis^1^, that was traditionally envisioned to be a treatment option in high-risk surgical candidates^2^.

However, the encouraging results derived from numerous randomized trials^3,4^ and observational registries^5^ corroborate TAVR as a reliable alternative to conventional surgical aortic valve replacement in high-risk and intermediate-risk patients and demonstrates a future potential even to moderate to mild risk patients^3,4,6–8^. Whilst initially TAVR was limited to a few first generation devices, the widespread use of this treatment option has prompted the refinement and improvement of the available prosthesis and the introduction of next-generation devices. Although these novel devices harbour potential significant technical advantages (e.g. smaller delivery sheets or ability to reposition), data directly comparing first and next generation devices are scarce. It seems likely that the technical advantages of the novel prosthesis combined with the increasing operator experience translate into an improved safety profile and reduced procedure related complications^9^. To date it is known that first generation devices harbour high rates of peri-procedural complications, such as stroke, vascular access site complications, valve malpositioning, and paravalvular regurgitation that impact survival. However, data comparing first and next generation devices are scarce, and it is still unknown if recent technological advances help to overcome the pitfalls of first generations prostheses. Overall, the new devices promise a prosperous future for this technique, as the new technical developments may allow us to expand its application and establish it as the main treatment option for symptomatic severe aortic stenosis in a broad spectrum of patients exceeding the initially selected population the inoperable high risk patients.

Aim of the present weighted meta-analysis was (1) to review pooled data on major adverse cardiovascular events from all currently available TAVR prosthesis in order to provide state-of-the-art insights regarding the safety profile of TAVR and (2) to assess the transition from first to next generation TAVR devices by directly comparing both in regard of procedure related complications.

## Methods

The utilized search strategy, study selection, data extraction, and analysis were performed according to the PRISMA guidelines for systematic reviews and meta-analyses^10^.

### Search strategy

Three authors (M.P.W., M.Z. and F.H.) independently and systematically searched PUBMED, SCOPUS, Cochrane Central Register of Controlled Trials, Cochrane Database of Systematic Reviews and LILACs for eligible trials from January 2000 to October 2017. In order to prevent potential publication bias, trial registries (https://www.who.int/trialsearch/Default.aspx, the WHO International Clinical Trials Registry Platform, and https://www.clinicaltrials.gov) were additionally screened for on-going and completed trials. The retrieval strategy was based on the combination of disease, therapy and study design using “AND” and “OR”. The following search terms were combined as keywords or MeSH terms: “aortic valve stenosis” or “aortic valve disease” with “transcatheter” or “transfemoral”or “transapical” or “TAVR” or “TAVI”. The search was limited to prospectively collected data in adults published in English.

### Study selection and data extraction

All published studies investigating transcatheter aortic valve replacement were identified. M.W and G.G. screened titles and/or abstracts for inclusion. Afterwards, all potentially suitable manuscripts were reviewed for final eligibility. Duplicates were identified using the reference management software EndNote X7 (Thomson Reuters, NY, USA) and excluded. In the present analysis, only prospective trials or registries were accepted. Full-texts of all includable trials were obtained and three investigators (M.P.W., A.B. and M.Z.) independently assessed study eligibility. In a second step the outcome variables were extracted. The following details were recorded for each study: first author, title, PubMed Identifier (PMID), study design, patient characteristics, inclusion criteria, valve type and delivery route, echocardiographic data, and funding.

### Definitions and interventions

Studies were only eligible if participants were prospectively included and if the studies reported sizing and implantation strategies as well as complications and mortality rates for approved TAVR valves specifying valve type and time point of assessment. Studies investigating rare aetiologies of aortic valve disease (valve thrombosis, bicuspid valves), other indications than conventional TAVR (valve-in-valve procedure, bail-out procedures, non-aortic position), and cerebral protection devices were not eligible for inclusion. Studies investigating more than one valve type were only included, if a separate analysis of every valve type was available.

### Outcomes and measurements

The primary parameters were the number/ratios of patients experiencing a procedure related transient ischemic attack (TIA), stroke, myocardial infarction, coronary obstruction, vascular complication, bleeding complication or death obtained at discharge/30 days. Additionally, all related surgical/interventional procedures (pacemaker implantation, cardiac re-intervention, surgical replacement and balloon post-dilation) were recorded.

### Study quality assessment

M.P.W. and G.G. evaluated all included studies for methodological quality according to the Cochrane Handbook for Systematic Reviews of Interventions 5.1.0.^11^. Studies with “inadequate” methodology were excluded. Disagreements were resolved by consensus.

## Results

**Figure 1:**
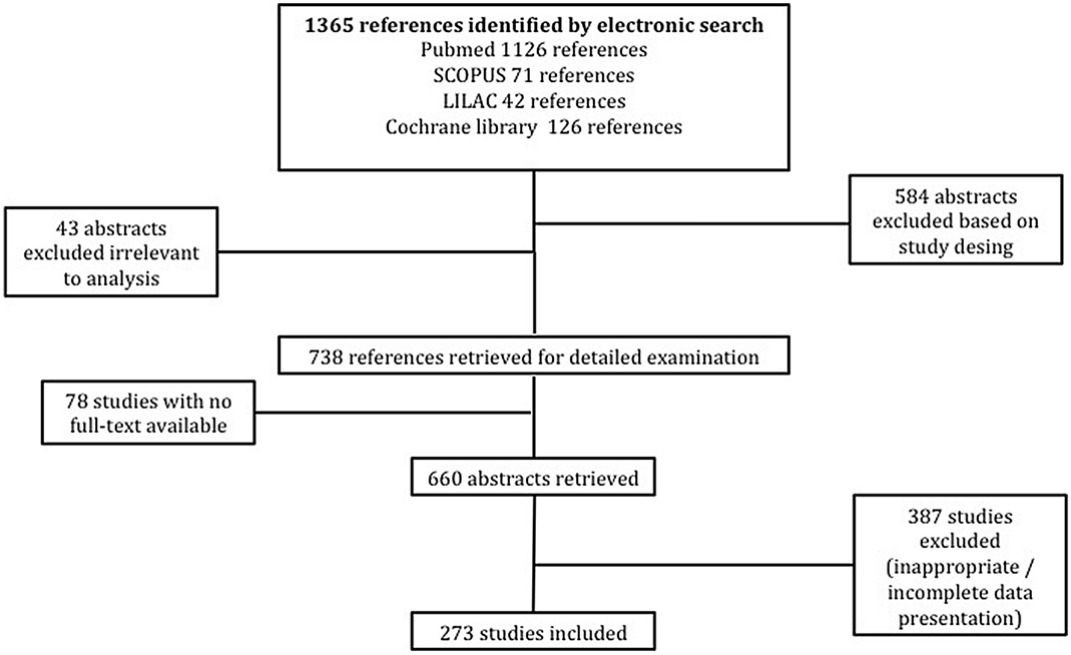
Flow diagram of study selection.

The predefined retrieval strategy on PUBMED, SCOPUS, Cochrane Central Register of Controlled Trials and LILAC yielded 1,365 citations that were initially evaluated for eligibility upon title and abstract level (Fig. 1). After exclusion of studies not complying with the inclusion criteria, 273 articles reporting major adverse cardiac events or complications related to TAVR procedures in 68,193 patients were included and are reported in detail in the Supplemental File 1 (references 29–301). Inter-readers agreement was satisfactory (Kappa coefficient 0.97). Overall, 108 studies in 27,707 patients reported measurements for the CoreValve, 48 studies with 14,060 patients for the SAPIEN XT valve, 48 studies with 13,831 patients for the SAPIEN valve, 22 studies with 6,834 patients for the SAPIEN 3 valve, 16 studies with 2,336 patients for the LOTUS Valve, 10 studies with 1,047 patients for the Direct Flow Medical Transcatheter valve, 4 studies with 133 patients for the ACURATE neo valve, 4 studies with 462 patients for the Portico valve, 4 studies with 1,425 patients for the Evolut R valve, 3 studies with 101 patients for the Engager valve, 2 studies with 207 patients for the JenaValve and 2 studies with 50 patients for die ACURATE TA (Table 1).

**Table 1:** Number of studies reporting complications according to valve type.

Valve type	No. studies	No. patients
CoreValve	108	27,707
SAPIEN XT	48	14,060
SAPIEN	48	13,831
SAPIEN 3	22	6,834
LotusValve	16	2,336
Direct Flow Medical	10	1,047
ACURATE Neo	4	133
Portico	4	462
Evolut R	4	1,425
Engager	3	101
JenaValve	2	207
ACURATE TA	2	50

### Range of major adverse cardiac events according to prosthesis

**Figure 2:**
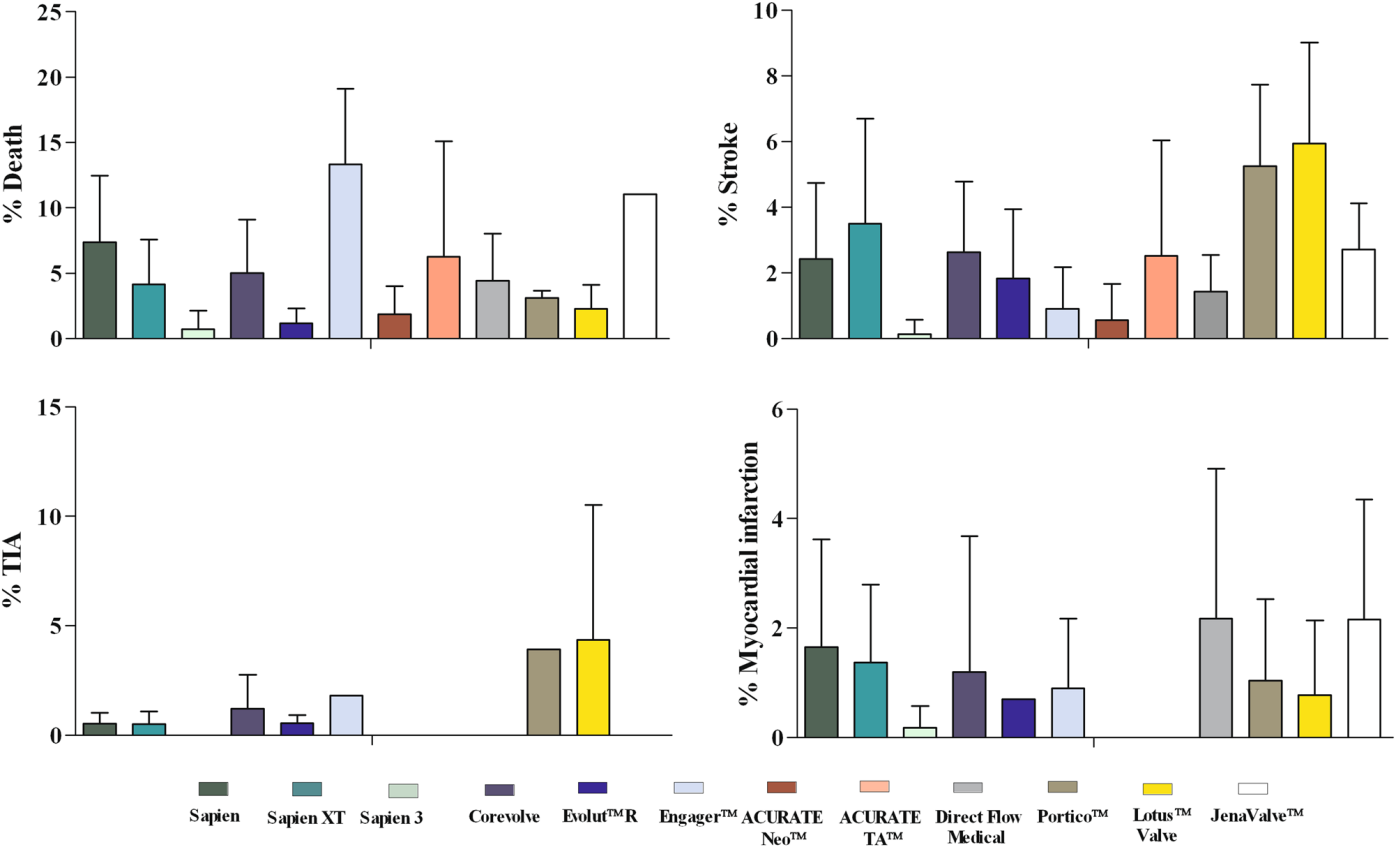
Occurrence of major adverse cardiac events according to implanted prosthesis type.

In total, 245 studies reported death rates, 216 studies the incidence of peri-interventional stroke, 163 studies the incidence acute myocardial infarction, and 44 studies the incidence transitory ischemic attacks (Table 2). Peri-procedural death rates ranged from 13.30 ± 5.80% (Engager) to 0.69 ± 1.44% (SAPIEN 3). Acute myocardial infarction was relatively uncommon with incidence rates ranging from 2.17 ± 2.74% (Direct Flow Medical) to 0.17 ± 0.40% (SAPIEN 3). Overall our analysis revealed the highest rates of neurological complications like TIA (4.35 ± 6.15%) and stroke (5.94 ± 3.07%) in patients receiving the Lotus valve (Table 2, Fig. 2).

**Table 2:** Occurrence of major adverse cardiac events after TAVR according to the available prosthesis.

Valve	Death (%)	N_Studies_/N_Patients_	TIA (%)	N_Studies_/N_Patients_	Stroke (%)	N_Studies_/N_Patients_	MI (%)	N_Studies_/N_Patient_
SAPIEN	7.35 ± 5.09	42/11,413	0.52 ± 04.9	5/1,086	2.41 ± 2.33	38/10,934	1.65 ± 1.97	20/8,145
SAPIEN XT	4.14 ± 3.41	37/13,323	0.50 ± 0.58	10/8,501	3.49 ± 3.21	41/13,068	1.36 ± 1.43	23/9,857
SAPIEN 3	0.69 ± 1.44	25/5,571	n.a	0/0	0.13 ± 0.44	25/5,571	0.17 ± 0.40	19/4,582
CoreValve	5.01 ± 4.09	97/26,390	1.20 ± 1.55	19/8,294	2.62 ± 2.15	70/21,805	1.19 ± 2.48	65/23,897
LotusValve	2.27 ± 1.85	16/2,383	4.35 ± 6.15	2/34	5.94 ± 3.07	12/1975	0.77 ± 1.36	13/1861
ACURATE Neo	1.85 ± 2.15	4/173	0.00 ± 0.00	2/69	0.55 ± 1.10	4/162	0.00 ± 0.00	4/162
ACURATE TA	6.25 ± 8.83	2/50	0.00	1/10	2.50 ± 3.53	2/50	0.00	1/40
Portico	3.1 ± 0.56	4/462	3.90	1/102	5.24 ± 2.49	5/484	1.04 ± 1.49	5/484
Direct flow medical	4.40 ± 3.61	8/989	n.a	0/0	1.42 ± 1.12	9/917	2.17 ± 2.74	8/745
JenaValve	11.10 ± 0.0	2/207	0.00	1/180	2.70 ± 1.41	2/207	2.15 ± 2.19	2/207
Evolut R	1.15 ± 1.13	5/1,499	0.54 ± 0.36	2/1,279	1.82 ± 2.11	5/1,499	0.70	1/1,038
Engager	13.30 ± 5.80	3/101	1.80	1/61	0.90 ± 1.27	2/71	0.90 ± 1.27	2/71

*TIA* transitory ischemic attack, *MI* myocardial infarction.

### Procedure-related complications according to prosthesis

241 studies reported the incidence rates for post-interventional pacemaker dependency, 126 studies the incidence of moderate/severe paravalvular regurgitation, 93 studies the rates for the need of surgical valve replacement, 55 studies the rates for the need of balloon post-dilation, 47 studies the incidence of coronary obstruction and 29 studies the rates for the need of cardiac re- intervention. Post-procedural pacemaker dependency is a well reported complication after TAVR, with the highest incidence rates in patients receiving the Lotus valve (28.64 ± 6.62%). Overall, we found that self-expanding TAVR prostheses were significantly more often associated with post-procedural conduction disturbances as compared to balloon-expandable prostheses (23.83 ± 10.66% vs. 8.79 ± 4.36, p < 0.001).

We pooled available data for moderate and severe paravalvular leakage of the different devices and calculated the combined incidence of moderate/severe regurgitation. We observed a wide range of prevalence with no reported leakage in the Accurate valves (Neo and TA), and a high prevalence of 15.70 ± 9.64% for the CoreValve. Out of all investigated TAVR prosthesis, in four valves (Direct Flow Medical, ACURATE TA, JenaValve, Lotus Valve) data on balloon post-dilation is unavailable. The other prostheses reported a wide range of incidence rates from 5.33 ± 8.16% (SAPIEN 3) to 33.63 ± 8.97% (ACURATE Neo). Cardiac re-interventions and surgical replacement were only infrequently reported and relatively rare (Table 3, Fig. 2).

**Table 3:** Occurrence of TAVR implantation related complications according to the available prosthesis.

Valve	PPI (% )	N_Studies_/N_Patients_	PVL II-III	N_Studies_/N_Patients_	Card. reint. (%)	N_Studies_/N_Patients_	Surg. replacement (%)	N_Studies_/N_Patients_	Balloon post-dilation (%)	N_Studies_/N_Patients_	Cor. Obs. (%)	N_Studies_/N_Patients_
SAPIEN	7.09 ± 4.36	37/10,768	11.02 ± 7.22	12/3,435	2.40	1/419	3.04 ± 3.57	17/3,711	6.99 ± 9.70	12/1731	1.41 ± 2.11	7/826
SAPIEN XT	8.59 ± 3.41	40/13,008	4.89 ± 3.53	16/1934	2.65 ± 0.21	2/289	1.74 ± 1.40	16/2,937	15.80 ± 17.81	11/1,190	2.00 ± 1.21	4/900
SAPIEN 3	11.51 ± 4.48	26/5,948	2.04 ± 2.61	22/4,260	1.40	1/815	0.87 ± 2.17	10/3,711	5.33 ± 8.16	9/983	0.00 ± 0.00	8/952
CoreValve	25.38 ± 10.32	90/26,188	15.70 ± 9.64	45/12,481	1.15 ± 0.21	2/1,485	1.96 ± 2.90	21/12,673	24.25 ± 12.80	17/9,061	0.35 ± 0.64	11/5,127
LotusValve	28.64 ± 6.62	19/2,935	0.65 ± 1.27	11/1571	0.12/0.30	12/923	0.51 ± 1.44	13/942	n.a	0/0	0.48 ± 0.44	5/738
ACURATE Neo	7.10 ± 2.82	3/158	0.00 ± 0.00	4/132	3.40	1/89	0.275 ± 0.55	4/162	33.63 ± 8.97	3/44	0.00 ± 0.00	4/133
ACURATE TA	3.75 ± 5.30	2/50	0.00 ± 0.00	2/50	n.a	0/0	0.00	1/10	n.a	0/0	n.a	0/0
Portico	10.04 ± 3.77	5,484	2.38 ± 2.17	4/262	n.a	0/0	0.00	1/22	9.10	1/22	0.00	1/102
Direct flow medical	13.98 ± 6.79	9/960	1.25 ± 1.77	2/218	1.75 ± 1.65	6/512	6.68 ± 5.80	4/422	n.a	0/0	1.60 ± 2.62	2/281
JenaValve	14.40	1/180	0.00	1/27	n.a	0/0	2.80	1/180	n.a	0/0	0.00	1/180
Evolut R	14.08 ± 2.58	5/1,499	4.00 ± 3.42	5/1,499	0.150 ± 0.30	4/1,425	0.00 ± 0.00	2/146	21.70	1/60	0.10 ± 0.20	4/1,425
Engager	19.20 ± 8.83	3/101	0.00	1/30	n.a	0/0	10	1/30	3.30	1/30	n.a	0/0

*PPI* new permanent pacemaker implantation, *PVL* paravalvular leakage, *Card.Reint.* cardiac re-intervention, *Cor.Obs.* coronary obstruction.

Data on vascular complications or bleeding could be extracted for all available TAVR prosthesis.

We observed that the SAPIEN 3 the lowest rates of vascular complications (3.75 ± 3.63%) whilst for the SAPIEN XT the highest rate vascular complications (12.75 ± 6.82%) have been reported (Table 4). Post- interventional bleeding was common with incidences ranging from 3.90 ± 4.89% (EvolutR) to 11.49 ± 6.78% (CoreValve). (Table 4, Fig. 2).

**Table 4:** Occurrence of TAVR implantation related vascular access site complications.

Valve	Vasc. compl. (%)	N_Studies_/N_Patients_	Bleeding (%)	N_Studies_/N_Patients_
SAPIEN	12.69 ± 8.40	25/97	9.11 ± 5.96	19/7,746
SAPIEN XT	12.75 ± 6.82	31/4,630	7.48 ± 4.94	30/11,382
SAPIEN 3	3.75 ± 3.63%	24/3,623	6.29 ± 6.18	20/5,033
CoreValve	11.21 ± 6.79	59/17,997	11.49 ± 6.78	39/16,009
LotusValve	9.10	1/11	9.67 ± 4.88	13/2,128
ACURATE Neo	1.35 ± 1.66	4/167	6.55 ± 6.29	4/173
ACURATE TA	2.50	1/40	0.00	1/40
Portico	12.00 ± 6.88	4/462	13.10 ± 1.06	3/181
Direct flow medical	3.80 ± 4.70	6/699	5.10 ± 2.91	6/856
JenaValve	8.30	1/180	7.15 ± 4.88	2/207
Evolut R	6.92 ± 5.00	5/1,499	3.90 ± 4.89	5/1,499
Engager	n.a	0/0	1.70	1/61

### Evolution of TAVR- related complications according to prosthesis generation

**Figure 3:**
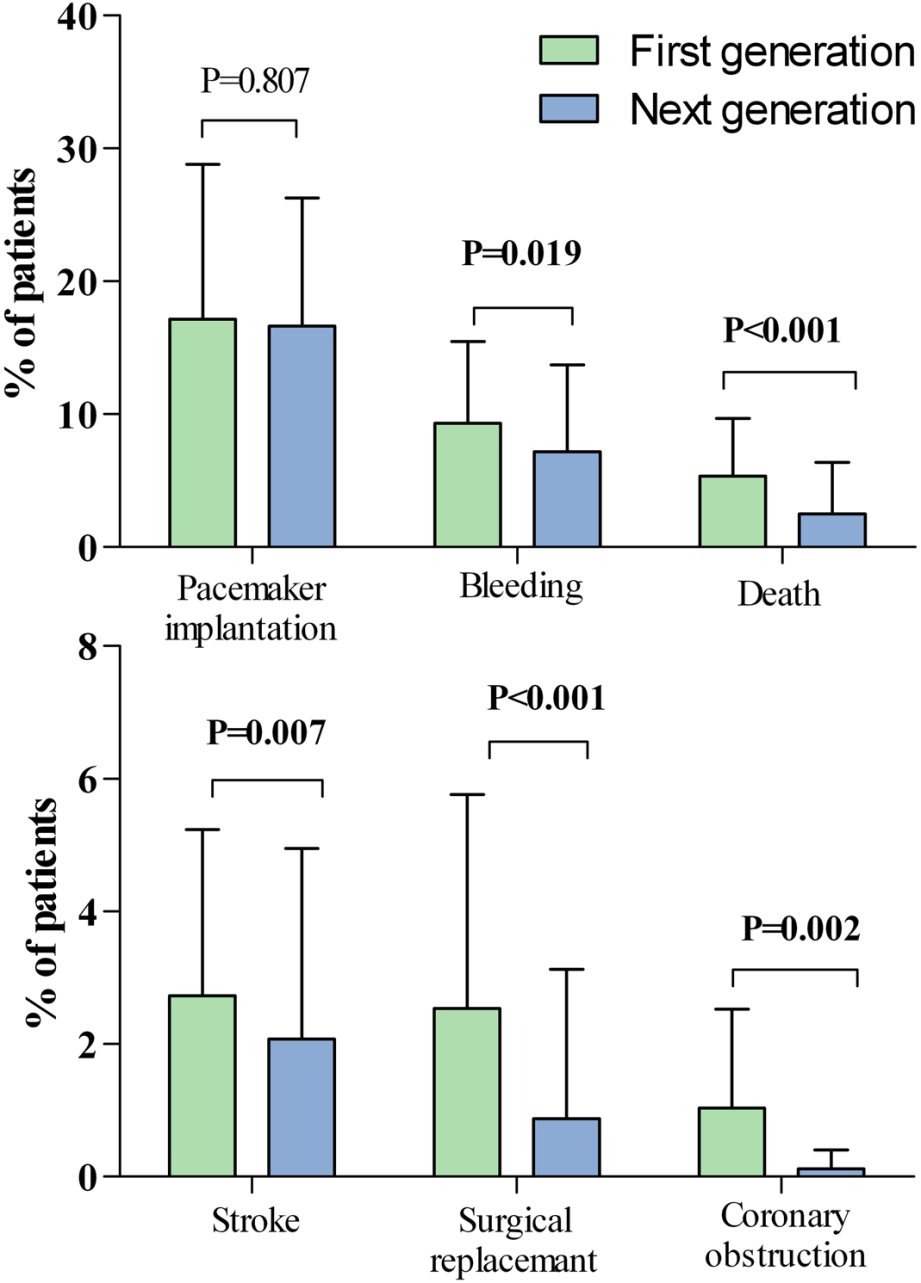
Occurrence of major adverse cardiac according to first and second generation devices.

To examine the influence of prosthesis generation on clinical outcome, we pooled available data of first generation devices (Edwards SAPIEN and SAPIEN XT, JenaValve, Direct flow Medical and CoreValve) and compared the outcomes with those from next generation devices (i.e. ACURATE Neo, Portico, Evolut R, Lotus Valve and SAPIEN 3). We observed that over time with the implementation of next generation devices the rate of peri-interventional stroke (2.09 ± 2.93 vs. 2.73 ± 2.49%, p = 0.009) and acute myocardial infarction (0.45 ± 0.97 vs. 1.39 ± 2.22%, P < 0.001) significantly decreased. Furthermore, the pooled analyses revealed a significant decrease in peri-interventional mortality in patients with a new TAVR prosthesis (1.47 ± 1.73 vs. 5.41 ± 4.35%; p < 0.001). With respect to TAVR-related complications, patients with next generation devices significantly less often experienced the need for a cardiac re-intervention (0.38 ± 0.86 vs. 1.86 ± 1.28%; p = 0.002), the occurrence of coronary obstructions (0.13 ± 0.29 vs. 0.99 ± 1.47%; p = 0.0049) and the necessity of surgical replacement (0.54 ± 1.55 vs. 2.54 ± 3.19%; p = 0.001). Furthermore, access site complications like bleeding (7.48 ± 6.62 vs. 9.27 ± 6.12%; p = 0.049) and vascular complications (5.42 ± 4.75 vs. 11.52 ± 7.23%; p < 0.001) were less common in newer generation devices. The pooled analysis revealed that the next generation devices showed significantly lower rate of moderate to severe paravalvular regurgitation as compared to their first generation antecessors (1.75 ± 2.43 vs. 12.39 ± 9.38%; p < 0.001) (Table 5, Fig. 3).

**Table 5:** TAVR procedure related complications according to first and next generation TAVR prosthesis.

Complication /Related procedure	First generation TAVR	N_Studies_/N_Patients_	Next generation TAVR	N_Studies_/N_Patients_	*p* value
PM Implantation (%)	17.12 ± 11.64	177/51,104	16.99 ± 9.66	58/11,024	0.681
PVL II–III	12.39 ± 9.38	74/17,877	1.75 ± 2.43	48/7,942	**< 0.000**
TIA (%)	0.87 ± 1.24	35/18,061	1.95 ± 3.29	7/1,484	0.972
Stroke (%)	2.73 ± 2.49	160/46,931	2.09 ± 2.93	52/9,893	**0.009**
Myocardial infarction (%)	1.39 ± 2.22	118/42,851	0.45 ± 0.97	42/8,127	**< 0.000**
Coronary obstruction (%)	0.99 ± 1.47	25/7,314	0.13 ± 0.29	22/3,350	**0.004**
Vascular complication (%)	11.51 ± 7.23	122/33,242	5.42 ± 4.75	51/7,904	**< 0.000**
Cardiac Reintervention (%)	1.86 ± 1.28	11/2,705	0.38 ± 0.866	18/3,252	**0.002**
Surgical replacement (%)	2.54 ± 3.19	59/20,096	0.54 ± 1.55	30/4,983	**< 0.000**
Balloon (%)	16.73 ± 15.15	40/11,982	12.83 ± 14.12	14/1,109	0.252
Bleeding (%)	9.27 ± 6.12	96/36,200	7.48 ± 6.62	45/9,014	**0.049**
Death (%)	5.41 ± 4.35	186/55,322	1.47 ± 1.73	54/10,088	**< 0.000**

Significant *p* values are indicated in bold.

*PM* pacemaker, *PVL* paravalvular leakage, *TIA* transitory ischemic attack.

## Discussion

In the present analysis, we provide a comprehensive overview on the current and past development regarding major adverse cardiac events and complications of TAVR extracted and pooled from the currently available literature encompassing a total of 68,193 TAVR implantations. All 273 studies included, were either prospective observational studies, national and device-specific registries, or randomized clinical trials. The principle aim of this study was to establish an overview on common complications related to the different TAVR devices and to pool the data from first and next generation devices, in order to compare their performance.

### Mortality after TAVR

Overall, our pooled analysis demonstrates a gradual improvement in peri-procedural mortality with next generation devices as compared with first generation devices. This may be attributed to various factors in the evolution of TAVR over the last decade. First, the technological advances leading to an improved hemodynamic performance and increased safety profile^12^. Secondly, a shift of risk starting with high risk patients in the advent of TAVR, now pushing the frontier to lower risk patient populations (class IIa, LoE B) with a known lower rate of complications and mortality^13^. And finally, not only the patient population but also the increasing operator experience seems to be a major determinant of improved outcome after TAVR. The PARTNER nonrandomized continued access registry (NRCA) of the PARTNER trial has investigated the influence of case selection and increasing case experience on mortality. Although there was no difference in STS score, there was a significant decline in mortality over time. In conclusion, this decrease is likely multifactorial as a result of strategic case selection, improved procedural techniques and increased site experience^14^.

### Neurologic outcomes after TAVR

Cerebrovascular events after TAVR procedures are serious complications, affecting patient survival with potential devastating neurological sequelae. These events are multifactorial and include embolic debris liberated from the native aortic valve, thrombi, bleeding and hemodynamic instability^15^.

Overall, prosthesis design has not been shown to influence the occurrence of cerebrovascular events^16^, however we found that patients with next generation devices a slight but statically significant lower rate of peri-procedural strokes as compared to patients with first generation devices. Whether this observation is attributable to improved valve architecture, is difficult to evaluate. In general, one can assume that acute (24 h) and subacute (< 30 days) events are more likely to be attributable to the TAVR procedure, whereas later events are more likely to be a sequela of comorbidities^17^. However, the timing of peri-procedural stroke is only infrequently reported and cannot be assessed in this analysis. Furthermore, data on optimal antiplatelet therapy is scarce and recommendations are based on opinions rather than on controlled randomized data^18^. The ACCF/AATS/SCAI/STS guidelines recommend dual antiplatelet treatment (DAPT) with aspirin and clopidogrel with no further recommendation on duration^19^. However, histopathologic studies revealed that at least 3 month DAPT are required for the complete endotheliazation of a TAVR prosthesis^20,21^. Although statistically significant our data show that the stroke rates only marginally decreased over time, which further underpins the unmet need of optimized antiplatelet and anticoagulation strategy therapy after TAVR.

### Need for pacemaker implantation after TAVR

Conduction disturbances are a common complication of the TAVR procedure, as it is (1) the consequence of mechanical compression exerted on the atrioventricular conduction tissue during predilation and prosthesis positioning, (2) protrusion of the prosthesis in the left ventricular outflow tract or (3) traumata by catheters and guidewires^21,22^. In our analysis, we observed in line previous data showing some valve types more frequently lead to permanent pacemaker implantation (PPI). The highest rate of PPI was seen for the Lotus Valve^23,24^. Although most next generation devices allow a high positioning with only minimal protrusion into the left ventricular outflow tract, we found no difference in PPI between first and next generation devices as we pooled the different expansion systems. Therefore the need of PPI is still highly attributable to the expansion system. The prognostic implications of this complication are noteworthy, as it is well evidenced that conduction disorders are associated with poor outcome, have a negative impact on LV function, and prolong hospital stay with increased procedural costs^25^.

### Coronary complications after TAVR

Coronary occlusion is a rare complication of the TAVR procedure with reported mortality rates of up to 50%^4,18,26^. Our pooled analysis showed that using next generation devices this complication is significantly less frequent, which is potentially attributable to the increasing operator experience. Furthermore, we found significantly decreased rates of peri-procedural acute myocardial infarctions in next generation devices, that suggests an advantages of next generations devices architecture with regard to protection of the coronary ostia during valve implantation. However, as mentioned above we can not exclude a significant impact of changed antiplatelet and anticoagulation strategy on AMI rates in our pooled analysis.

### Paravalvular regurgitation

Paravalvular leaks (PVL) are major determinant of hemodynamic performance of TAVR prostheses. Although often believed to be negligable, even mild PVL after TAVR has been shown to negatively influence survival after the procedure^27^. The PVL rate in first generation devices of up to 24% and the associated adverse impact on patient outcome served as powerful incentive to refine the design of TAVR prosthesis in order to reduce this complication. Beside interventional procedure (interventional closure, snare technique, balloon post-dilation), sealing mechanisms (i.e. skirts) at the lower part of the valve prosthesis have considerably helped to compensate irregular surfaces from the calcified native valve and to reduce PVL after TAVR implantation^28^. In our analysis, we found a dramatic decrease in moderate to severe PVL after TAVR implantation in next generation devices as compared to their predecessors. Overall this dramatic improvement is possibly linked to several factors such as, refined prosthesis design, refined valve architecture, refined imaging techniques with improved sizing and better delivery techniques with partially to fully repositionable prostheses.

### Access site complications related to the TAVR procedure

The incidence of major vascular complications has been reported to be up to 33% (19). To reduce this risk in patients with unfavourable iliofemoral anatomy alternative access routes have been proposed. Efforts have been made to miniaturize delivery systems and to refine available valves to a low crossing profile^21^. Overall, these efforts have led to a downsizing of the delivery systems from 24–25 French (Cribier-Edwards Valve) to the current 14–18 French. The downsizing of the devices has reduced the need of surgical access site interventions, simplify the procedure, increase the deliverability and reduce the risk of vascular complications. Taken together, we observed significantly reduced bleeding rates and a dramatic decrease in vascular complications for newer generation devices. This superior safety profile with regard to access site complications is an important prerequisite for an early discharge after the TAVR procedure being cost effective^29^, increases patient comfort, and the mental and emotional aspects that affect the recovery after the procedure^30^.

### Limitations

The present analysis harbours some important limitations: Firstly, when comparing first versus next generation prostheses regarding outcome and complications, we must not neglect steadily changing patients populations. Whilst first generation prosthesis were mainly implanted in high risk patients, next generation prostheses become more and more a treatment option for moderate risk patients. This patient population has a known lower rate of complications and a lower mortality rate. Secondly, in some next generation devices only limited data is available and real world outcome might not be comparable. A significant proportion of the included trials did not report data on STS or EuroSCORE, for this purpose a correction regarding the baseline risk profile was not feasible. Lastly, we only included studies that clearly reported complications.

## Conclusion

The present analysis proves for the first time, that the rapidly evolving TAVR technology translates into significantly reduced peri-procedural complications, paravalvular regurgitation and most importantly mortality. Overall, the advancements of next generation TAVR prosthesis have led to an impressive improvement in TAVR safety driven by refined case selection, improved procedural techniques and increased site experience.

## Supplementary information

Supplementary information.
