# Supplementary material for: Evolution of outcome and complications in TAVR: a meta-analysis of observational and randomized studies
Source: Sci Rep. 2020 Sep 23;10:15568. doi: 10.1038/s41598-020-72453-1 (PMC7511292; doi:10.1038/s41598-020-72453-1)
Supplement: Supplementary file 1 — Supplementary information. [file 41598_2020_72453_MOESM1_ESM.docx]

Evolution of Outcome and Complications in TAVR –

A Meta-Analysis of Observational and Randomized Studies

Max-Paul Winter MD,^1^ Philipp Bartko MD, PhD,^1^ Felix Hofer,^1^ Martin Zbiral,^1^ Achim Burger,^1^ Bahil Ghanim MD, PhD,^2^ Johannes Kastner MD, ^1^ Irene M. Lang MD, ^1^ Julia Mascherbauer MD, ^1^

Christian Hengstenberg MD,^1^ and Georg Goliasch MD, PhD^1^

^1^Department of Internal Medicine II, Medical University of Vienna, Vienna, Austria

^2^ Department of General and Thoracic Surgery, University Hospital Krems, Karl Landsteiner University of Health Sciences

Correspondence to: Georg Goliasch MD, PhD

Department of Internal Medicine II

Medical University of Vienna,

Waehringer Guertel 18-20

A-1090 Vienna/ Austria

Tel. ++43-1-40400-46060

Fax. ++43-1-40400-42160

Email: [georg.goliasch@meduniwien.ac.at](mailto:georg.goliasch@meduniwien.ac.at)

***Supplementary references***

29 Mollmann H, Walther T, Siqueira D et al. Transfemoral TAVI using the self-expanding ACURATE neo prosthesis: one-year outcomes of the multicentre "CE-approval cohort". EuroIntervention 2017;13:e1040-e1046.

30 Grube E, Van Mieghem NM, Bleiziffer S et al. Clinical Outcomes With a Repositionable Self-Expanding Transcatheter Aortic Valve Prosthesis: The International FORWARD Study. J Am Coll Cardiol 2017;70:845-853.

31 Misterski M, Puslecki M, Grygier M et al. Transapical aortic valve implantation using a Symetis Acurate self-expandable bioprosthesis: initial outcomes of 10 patients. Wideochir Inne Tech Maloinwazyjne 2017;12:172-177.

32 Falk V, Wohrle J, Hildick-Smith D et al. Safety and efficacy of a repositionable and fully retrievable aortic valve used in routine clinical practice: the RESPOND Study. Eur Heart J 2017;38:3359-3366.

33 Schulz E, Jabs A, Gori T et al. Transcatheter aortic valve implantation with the new-generation Evolut R: Comparison with CoreValve(R) in a single center cohort. Int J Cardiol Heart Vasc 2016;12:52-56.

34 Wendler O, Schymik G, Treede H et al. SOURCE 3: 1-year outcomes post-transcatheter aortic valve implantation using the latest generation of the balloon-expandable transcatheter heart valve. Eur Heart J 2017;38:2717-2726.

35 Giannini C, De Carlo M, Tamburino C et al. Transcathether aortic valve implantation with the new repositionable self-expandable Evolut R versus CoreValve system: A case-matched comparison. Int J Cardiol 2017;243:126-131.

36 Romano M, Frank D, Cocchieri R et al. Transaortic transcatheter aortic valve implantation using SAPIEN XT or SAPIEN 3 valves in the ROUTE registry. Interact Cardiovasc Thorac Surg 2017;25:757-764.

37 Taramasso M, Denegri A, Kuwata S et al. Feasibility and safety of transfemoral sheathless portico aortic valve implantation: Preliminary results in a single center experience. Catheter Cardiovasc Interv 2018;91:533-539.

38 Rogers T, Steinvil A, Buchanan K et al. Contemporary transcatheter aortic valve replacement with third-generation balloon-expandable versus self-expanding devices. J Interv Cardiol 2017;30:356-361.

39 Bleiziffer S, Bosmans J, Brecker S et al. Insights on mid-term TAVR performance: 3-year clinical and echocardiographic results from the CoreValve ADVANCE study. Clin Res Cardiol 2017;106:784-795.

40 Kallinikou Z, Berger A, Ruchat P et al. Transcutaneous aortic valve implantation using the carotid artery access: Feasibility and clinical outcomes. Arch Cardiovasc Dis 2017;110:389-394.

41 Di Martino LFM, Soliman OII, van Gils L et al. Relation between calcium burden, echocardiographic stent frame eccentricity and paravalvular leakage after corevalve transcatheter aortic valve implantation. Eur Heart J Cardiovasc Imaging 2017;18:648-653.

42 Sawa Y, Torikai K, Kobayashi J et al. Midterm Outcomes With a Self-Expandable Transcatheter Heart Valve in Japanese Patients With Symptomatic Severe Aortic Stenosis. Circ J 2017;81:1108-1115.

43 Krackhardt F, Kherad B, Krisper M, Pieske B, Laule M, Tschope C. Low permanent pacemaker rates following Lotus device implantation for transcatheter aortic valve replacement due to modified implantation protocol. Cardiol J 2017;24:250-258.

44 O'Hair DP, Bajwa TK, Chetcuti SJ et al. One-Year Outcomes of Transcatheter Aortic Valve Replacement in Patients With End-Stage Renal Disease. Ann Thorac Surg 2017;103:1392-1398.

45 Popma JJ, Reardon MJ, Khabbaz K et al. Early Clinical Outcomes After Transcatheter Aortic Valve Replacement Using a Novel Self-Expanding Bioprosthesis in Patients With Severe Aortic Stenosis Who Are Suboptimal for Surgery: Results of the Evolut R U.S. Study. JACC Cardiovasc Interv 2017;10:268-275.

46 Montone RA, Testa L, Fraccaro C et al. Procedural and 30-day clinical outcomes following transcatheter aortic valve replacement with lotus valve: Results of the RELEVANT study. Catheter Cardiovasc Interv 2017;90:1206-1211.

47 Maeno Y, Abramowitz Y, Jilaihawi H et al. Optimal sizing for SAPIEN 3 transcatheter aortic valve replacement in patients with or without left ventricular outflow tract calcification. EuroIntervention 2017;12:e2177-e2185.

48 Perlman GY, Cheung A, Dumont E et al. Transcatheter aortic valve replacement with the Portico valve: one-year results of the early Canadian experience. EuroIntervention 2017;12:1653-1659.

49 Soliman OII, El Faquir N, Ren B et al. Comparison of valve performance of the mechanically expanding Lotus and the balloon-expanded SAPIEN3 transcatheter heart valves: an observational study with independent core laboratory analysis. Eur Heart J Cardiovasc Imaging 2018;19:157-167.

50 Aslan AN, Bastug S, Kasapkara HA et al. Transcatheter aortic valve implantation with the Edwards Sapien 3 valve: First experiences in Turkey. Turk Kardiyol Dern Ars 2016;44:663-669.

51 Yanagisawa R, Hayashida K, Yamada Y et al. Incidence, Predictors, and Mid-Term Outcomes of Possible Leaflet Thrombosis After TAVR. JACC Cardiovasc Imaging 2016.

52 Kaneko H, Hoelschermann F, Tambor G, Yoon SH, Neuss M, Butter C. Predictors of Paravalvular Regurgitation After Transcatheter Aortic Valve Implantation for Aortic Stenosis Using New-Generation Balloon-Expandable SAPIEN 3. Am J Cardiol 2017;119:618-622.

53 Bruschi G, Branny M, Schiltgen M et al. One-Year Outcomes of Transcatheter Aortic Valve Implantation Using the Direct Aortic Approach. Ann Thorac Surg 2017;103:1434-1440.

54 Sinning JM, Petronio AS, Van Mieghem N et al. Relation Between Clinical Best Practices and 6-Month Outcomes After Transcatheter Aortic Valve Implantation With CoreValve (from the ADVANCE II Study). Am J Cardiol 2017;119:84-90.

55 Kasapkara HA, Aslan AN, Ayhan H et al. Trans-subclavian aortic valve replacement with various bioprosthetic valves: Single-center experience. Turk Kardiyol Dern Ars 2016;44:582-589.

56 D'Ancona G, Agma HU, Ince H et al. Transcatheter aortic valve implantation with the direct flow medical prosthesis: Impact of native aortic valve calcification degree on outcomes. Catheter Cardiovasc Interv 2017;89:135-142.

57 Fiorina C, Bruschi G, Testa L et al. Transaxillary versus transaortic approach for transcatheter aortic valve implantation with CoreValve Revalving System: insights from multicenter experience. J Cardiovasc Surg (Torino) 2017;58:747-754.

58 Bagur R, Teefy PJ, Kiaii B, Diamantouros P, Chu MWA. First North American experience with the transfemoral ACURATE-neo(TM) self-expanding transcatheter aortic bioprosthesis. Catheter Cardiovasc Interv 2017;90:130-138.

59 Gonska B, Seeger J, Baarts J et al. The balloon-expandable Edwards Sapien 3 valve is superior to the self-expanding Medtronic CoreValve in patients with severe aortic stenosis undergoing transfemoral aortic valve implantation. J Cardiol 2017;69:877-882.

60 Yoon SH, Lefevre T, Ahn JM et al. Transcatheter Aortic Valve Replacement With Early- and New-Generation Devices in Bicuspid Aortic Valve Stenosis. J Am Coll Cardiol 2016;68:1195-1205.

61 Petzina R, Lutter G, Wolf C et al. Transaortic transcatheter aortic valve implantation: experience from the Kiel study. Interact Cardiovasc Thorac Surg 2017;24:55-62.

62 Attizzani GF, Ohno Y, Latib A et al. Age-Related Differences in 1- and 12-Month Outcomes in Patients Undergoing Transcatheter Aortic Valve Implantation (from a Large Multicenter Data Repository). Am J Cardiol 2016;118:1024-30.

63 Manoharan G, Linke A, Moellmann H et al. Multicentre clinical study evaluating a novel resheathable annular functioning self-expanding transcatheter aortic valve system: safety and performance results at 30 days with the Portico system. EuroIntervention 2016;12:768-74.

64 Wohrle J, Gonska B, Rodewald C, Seeger J, Scharnbeck D, Rottbauer W. Transfemoral aortic valve implantation with the repositionable Lotus valve for treatment of patients with symptomatic severe aortic stenosis: results from a single-centre experience. EuroIntervention 2016;12:760-7.

65 Eksik A, Yildirim A, Gul M et al. Comparison of Edwards Sapien XT versus Lotus Valve Devices in Terms of Electrophysiological Study Parameters in Patients Undergoing TAVI. Pacing Clin Electrophysiol 2016;39:1132-1140.

66 Kong WK, van Rosendael PJ, van der Kley F et al. Impact of Different Iterations of Devices and Degree of Aortic Valve Calcium on Paravalvular Regurgitation After Transcatheter Aortic Valve Implantation. Am J Cardiol 2016;118:567-71.

67 Little SH, Oh JK, Gillam L et al. Self-Expanding Transcatheter Aortic Valve Replacement Versus Surgical Valve Replacement in Patients at High Risk for Surgery: A Study of Echocardiographic Change and Risk Prediction. Circ Cardiovasc Interv 2016;9.

68 Dauerman HL, Reardon MJ, Popma JJ et al. Early Recovery of Left Ventricular Systolic Function After CoreValve Transcatheter Aortic Valve Replacement. Circ Cardiovasc Interv 2016;9.

69 Vahanian A, Urena M, Walther T et al. Thirty-day outcomes in patients at intermediate risk for surgery from the SAPIEN 3 European approval trial. EuroIntervention 2016;12:e235-43.

70 Bocksch W, Grossmann B, Geisler T et al. Clinical outcome and paravalvular leakage of the new balloon-expandable Edwards Sapien 3 valve in comparison to its predecessor model (Edwards Sapien XT) in patients undergoing transfemoral aortic valve replacement. Catheter Cardiovasc Interv 2016;88:466-75.

71 Yoon SH, Ahn JM, Hayashida K et al. Clinical Outcomes Following Transcatheter Aortic Valve Replacement in Asian Population. JACC Cardiovasc Interv 2016;9:926-33.

72 Husser O, Pellegrini C, Kessler T et al. Predictors of Permanent Pacemaker Implantations and New-Onset Conduction Abnormalities With the SAPIEN 3 Balloon-Expandable Transcatheter Heart Valve. JACC Cardiovasc Interv 2016;9:244-254.

73 Lefevre T, Colombo A, Tchetche D et al. Prospective Multicenter Evaluation of the Direct Flow Medical Transcatheter Aortic Valve System: 12-Month Outcomes of the Evaluation of the Direct Flow Medical Percutaneous Aortic Valve 18F System for the Treatment of Patients With Severe Aortic Stenosis (DISCOVER) Study. JACC Cardiovasc Interv 2016;9:68-75.

74 Barbanti M, Schiltgen M, Verdoliva S et al. Three-Year Outcomes of Transcatheter Aortic Valve Implantation in Patients With Varying Levels of Surgical Risk (from the CoreValve ADVANCE Study). Am J Cardiol 2016;117:820-7.

75 Del Trigo M, Dahou A, Webb JG et al. Self-expanding Portico Valve Versus Balloon-expandable SAPIEN XT Valve in Patients With Small Aortic Annuli: Comparison of Hemodynamic Performance. Rev Esp Cardiol (Engl Ed) 2016;69:501-8.

76 Husser O, Pellegrini C, Kessler T et al. Outcomes After Transcatheter Aortic Valve Replacement Using a Novel Balloon-Expandable Transcatheter Heart Valve: A Single-Center Experience. JACC Cardiovasc Interv 2015;8:1809-16.

77 Binder RK, Stortecky S, Heg D et al. Procedural Results and Clinical Outcomes of Transcatheter Aortic Valve Implantation in Switzerland: An Observational Cohort Study of Sapien 3 Versus Sapien XT Transcatheter Heart Valves. Circ Cardiovasc Interv 2015;8.

78 Conte JV, Gleason TG, Resar JR et al. Transcatheter or Surgical Aortic Valve Replacement in Patients With Prior Coronary Artery Bypass Grafting. Ann Thorac Surg 2016;101:72-9; discussion 79.

79 Zembala M, Hawranek M, Waclawski J et al. Symetis Acurate Neo transfemoral aortic bioprosthesis - initial Polish experience. Kardiol Pol 2016;74:206-12.

80 Philipsen TE, Collas VM, Rodrigus IE et al. Brachiocephalic artery access in transcatheter aortic valve implantation: a valuable alternative: 3-year institutional experience. Interact Cardiovasc Thorac Surg 2015;21:734-40.

81 Canadyova J, Mokracek A, Pesl L, Kurfirst V, Sulda M. Short-term and medium-term outcomes of transapical aortic valve implantation as a single-strategy approach: one center's experience. Kardiochir Torakochirurgia Pol 2015;12:95-102.

82 Manoharan G, Walton AS, Brecker SJ et al. Treatment of Symptomatic Severe Aortic Stenosis With a Novel Resheathable Supra-Annular Self-Expanding Transcatheter Aortic Valve System. JACC Cardiovasc Interv 2015;8:1359-67.

83 Abdel-Wahab M, Neumann FJ, Mehilli J et al. 1-Year Outcomes After Transcatheter Aortic Valve Replacement With Balloon-Expandable Versus Self-Expandable Valves: Results From the CHOICE Randomized Clinical Trial. J Am Coll Cardiol 2015;66:791-800.

84 Salaun E, Jacquier A, Theron A et al. Value of CMR in quantification of paravalvular aortic regurgitation after TAVI. Eur Heart J Cardiovasc Imaging 2016;17:41-50.

85 Di Martino LF, Vletter WB, Ren B et al. Prediction of paravalvular leakage after transcatheter aortic valve implantation. Int J Cardiovasc Imaging 2015;31:1461-8.

86 Barbanti M, Petronio AS, Ettori F et al. 5-Year Outcomes After Transcatheter Aortic Valve Implantation With CoreValve Prosthesis. JACC Cardiovasc Interv 2015;8:1084-1091.

87 Gooley RP, Talman AH, Cameron JD, Lockwood SM, Meredith IT. Comparison of Self-Expanding and Mechanically Expanded Transcatheter Aortic Valve Prostheses. JACC Cardiovasc Interv 2015;8:962-71.

88 Reardon MJ, Adams DH, Kleiman NS et al. 2-Year Outcomes in Patients Undergoing Surgical or Self-Expanding Transcatheter Aortic Valve Replacement. J Am Coll Cardiol 2015;66:113-21.

89 Wohrle J, Gonska B, Rodewald C et al. Transfemoral aortic valve implantation with the repositionable Lotus valve compared with the balloon-expandable Edwards Sapien 3 valve. Int J Cardiol 2015;195:171-5.

90 Yang TH, Webb JG, Blanke P et al. Incidence and severity of paravalvular aortic regurgitation with multidetector computed tomography nominal area oversizing or undersizing after transcatheter heart valve replacement with the Sapien 3: a comparison with the Sapien XT. JACC Cardiovasc Interv 2015;8:462-471.

91 Maeda K, Kuratani T, Torikai K et al. New Self-Expanding Transcatheter Aortic Valve Device for Transfemoral Implantation- Early Results of the First-in-Asia Implantation of the ACURATE Neo/TF(TM) System. Circ J 2015;79:1037-43.

92 Kempfert J, Meyer A, Kim WK et al. Comparison of two valve systems for transapical aortic valve implantation: a propensity score-matched analysis. Eur J Cardiothorac Surg 2016;49:486-92.

93 Webb J, Gerosa G, Lefevre T et al. Multicenter evaluation of a next-generation balloon-expandable transcatheter aortic valve. J Am Coll Cardiol 2014;64:2235-43.

94 Tarantini G, Mojoli M, Purita P et al. Unravelling the (arte)fact of increased pacemaker rate with the Edwards SAPIEN 3 valve. EuroIntervention 2015;11:343-50.

95 Kodali S, Pibarot P, Douglas PS et al. Paravalvular regurgitation after transcatheter aortic valve replacement with the Edwards sapien valve in the PARTNER trial: characterizing patients and impact on outcomes. Eur Heart J 2015;36:449-56.

96 Meredith Am IT, Walters DL, Dumonteil N et al. Transcatheter aortic valve replacement for severe symptomatic aortic stenosis using a repositionable valve system: 30-day primary endpoint results from the REPRISE II study. J Am Coll Cardiol 2014;64:1339-48.

97 Reuthebuch O, Inderbitzin DT, Ruter F et al. Single-center experience and short-term outcome with the JenaValve: a second-generation transapical transcatheter aortic valve implantation device. Innovations (Phila) 2014;9:368-74; discussion 374.

98 Barbanti M, Petronio AS, Capodanno D et al. Impact of balloon post-dilation on clinical outcomes after transcatheter aortic valve replacement with the self-expanding CoreValve prosthesis. JACC Cardiovasc Interv 2014;7:1014-21.

99 Tarsia G, Smaldone C, Viceconte NG et al. Lower cardiovascular mortality with Medtronic CoreValve versus Edwards SAPIEN in patients with aortic valve stenosis undergoing transcatheter aortic valve implantation. Int J Cardiol 2014;177:520-2.

100 Watanabe Y, Lefevre T, Arai T et al. Can we predict postprocedural paravalvular leak after Edwards SAPIEN transcatheter aortic valve implantation? Catheter Cardiovasc Interv 2015;86:144-51.

101 Amat-Santos IJ, Dahou A, Webb J et al. Comparison of hemodynamic performance of the balloon-expandable SAPIEN 3 versus SAPIEN XT transcatheter valve. Am J Cardiol 2014;114:1075-82.

102 Bozkurt E, Keles T, Durmaz T et al. Early outcomes of transcatheter aortic valve replacement in patients with severe aortic stenosis: single center experience. Postepy Kardiol Interwencyjnej 2014;10:84-90.

103 da Silva C, Sahlen A, Winter R et al. Hemodynamic outcomes of transcatheter aortic valve implantation with the CoreValve system: an early assessment. Clin Physiol Funct Imaging 2015;35:216-22.

104 Linke A, Wenaweser P, Gerckens U et al. Treatment of aortic stenosis with a self-expanding transcatheter valve: the International Multi-centre ADVANCE Study. Eur Heart J 2014;35:2672-84.

105 Abdel-Wahab M, Mehilli J, Frerker C et al. Comparison of balloon-expandable vs self-expandable valves in patients undergoing transcatheter aortic valve replacement: the CHOICE randomized clinical trial. JAMA 2014;311:1503-14.

106 Adams DH, Popma JJ, Reardon MJ et al. Transcatheter aortic-valve replacement with a self-expanding prosthesis. N Engl J Med 2014;370:1790-8.

107 Popma JJ, Adams DH, Reardon MJ et al. Transcatheter aortic valve replacement using a self-expanding bioprosthesis in patients with severe aortic stenosis at extreme risk for surgery. J Am Coll Cardiol 2014;63:1972-81.

108 Ayhan H, Durmaz T, Keles T et al. Improvement of right ventricular function with transcatheter aortic valve implantation. Scand Cardiovasc J 2014;48:184-8.

109 Walters DL, Sinhal A, Baron D et al. Initial experience with the balloon expandable Edwards-SAPIEN Transcatheter Heart Valve in Australia and New Zealand: the SOURCE ANZ registry: outcomes at 30 days and one year. Int J Cardiol 2014;170:406-12.

110 Genereux P, Cohen DJ, Williams MR et al. Bleeding complications after surgical aortic valve replacement compared with transcatheter aortic valve replacement: insights from the PARTNER I Trial (Placement of Aortic Transcatheter Valve). J Am Coll Cardiol 2014;63:1100-9.

111 Meredith IT, Worthley SG, Whitbourn RJ et al. Transfemoral aortic valve replacement with the repositionable Lotus Valve System in high surgical risk patients: the REPRISE I study. EuroIntervention 2014;9:1264-70.

112 Sinning JM, Adenauer V, Scheer AC et al. Doppler-based renal resistance index for the detection of acute kidney injury and the non-invasive evaluation of paravalvular aortic regurgitation after transcatheter aortic valve implantation. EuroIntervention 2014;9:1309-16.

113 De Carlo M, Giannini C, Fiorina C et al. Paravalvular leak after CoreValve implantation in the Italian Registry: predictors and impact on clinical outcome. Int J Cardiol 2013;168:5088-9.

114 Binder RK, Webb JG, Willson AB et al. The impact of integration of a multidetector computed tomography annulus area sizing algorithm on outcomes of transcatheter aortic valve replacement: a prospective, multicenter, controlled trial. J Am Coll Cardiol 2013;62:431-8.

115 Binder RK, Rodes-Cabau J, Wood DA et al. Transcatheter aortic valve replacement with the SAPIEN 3: a new balloon-expandable transcatheter heart valve. JACC Cardiovasc Interv 2013;6:293-300.

116 Nombela-Franco L, Ruel M, Radhakrishnan S et al. Comparison of hemodynamic performance of self-expandable CoreValve versus balloon-expandable Edwards SAPIEN aortic valves inserted by catheter for aortic stenosis. Am J Cardiol 2013;111:1026-33.

117 Seiffert M, Schnabel R, Conradi L et al. Predictors and outcomes after transcatheter aortic valve implantation using different approaches according to the valve academic research consortium definitions. Catheter Cardiovasc Interv 2013;82:640-52.

118 Verhoye JP, Lapeze J, Anselmi A, Donal E. Association of transaortic approach and transoesophageal echocardiography as the primary imaging technique for improved results in transcatheter valve implantation. Interact Cardiovasc Thorac Surg 2012;15:756-8.

119 Van Linden A, Kempfert J, Blumenstein J et al. Prosthesis-patient mismatch after transcatheter aortic valve implantation using the Edwards SAPIEN prosthesis. Thorac Cardiovasc Surg 2013;61:414-20.

120 Yared K, Garcia-Camarero T, Fernandez-Friera L et al. Impact of aortic regurgitation after transcatheter aortic valve implantation: results from the REVIVAL trial. JACC Cardiovasc Imaging 2012;5:469-77.

121 Kempfert J, Treede H, Rastan AJ et al. Transapical aortic valve implantation using a new self-expandable bioprosthesis (ACURATE TA): 6-month outcomes. Eur J Cardiothorac Surg 2013;43:52-6; discussion 57.

122 Modine T, Sudre A, Collet F et al. Transcutaneous aortic valve implantation using the axillary/subclavian access with patent left internal thoracic artery to left anterior descending artery: feasibility and early clinical outcomes. J Thorac Cardiovasc Surg 2012;144:1416-20.

123 Schultz CJ, Tzikas A, Moelker A et al. Correlates on MSCT of paravalvular aortic regurgitation after transcatheter aortic valve implantation using the Medtronic CoreValve prosthesis. Catheter Cardiovasc Interv 2011;78:446-55.

124 Sherif MA, Abdel-Wahab M, Beurich HW et al. Haemodynamic evaluation of aortic regurgitation after transcatheter aortic valve implantation using cardiovascular magnetic resonance. EuroIntervention 2011;7:57-63.

125 Munoz-Garcia AJ, Alonso-Briales JH, Jimenez-Navarro MF et al. Mechanisms, treatment and course of paravalvular aortic regurgitation after percutaneous implantation of the CoreValve aortic prosthesis. Int J Cardiol 2011;149:389-92.

126 Bruschi G, De Marco F, Fratto P et al. Alternative approaches for trans-catheter self-expanding aortic bioprosthetic valves implantation: single-center experience. Eur J Cardiothorac Surg 2011;39:e151-8.

127 Tamburino C, Capodanno D, Ramondo A et al. Incidence and predictors of early and late mortality after transcatheter aortic valve implantation in 663 patients with severe aortic stenosis. Circulation 2011;123:299-308.

128 Leon C, Suarez de Lezo J, Mesa D et al. Early development of leaks in the CoreValve percutaneous aortic valve prosthesis: echocardiographic assessment. Rev Esp Cardiol 2011;64:67-70.

129 Falk V, Walther T, Schwammenthal E et al. Transapical aortic valve implantation with a self-expanding anatomically oriented valve. Eur Heart J 2011;32:878-87.

130 Sherif MA, Abdel-Wahab M, Stocker B et al. Anatomic and procedural predictors of paravalvular aortic regurgitation after implantation of the Medtronic CoreValve bioprosthesis. J Am Coll Cardiol 2010;56:1623-9.

131 Attias D, Himbert D, Ducrocq G et al. Immediate and mid-term results of transfemoral aortic valve implantation using either the Edwards Sapien transcatheter heart valve or the Medtronic CoreValve System in high-risk patients with aortic stenosis. Arch Cardiovasc Dis 2010;103:236-45.

132 Modine T, Obadia JF, Choukroun E et al. Transcutaneous aortic valve implantation using the axillary/subclavian access: feasibility and early clinical outcomes. J Thorac Cardiovasc Surg 2011;141:487-91, 491 e1.

133 Jilaihawi H, Chin D, Spyt T et al. Prosthesis-patient mismatch after transcatheter aortic valve implantation with the Medtronic-Corevalve bioprosthesis. Eur Heart J 2010;31:857-64.

134 Al-Attar N, Himbert D, Descoutures F et al. Transcatheter aortic valve implantation: selection strategy is crucial for outcome. Ann Thorac Surg 2009;87:1757-62; discussion 1762-3.

135 Rodes-Cabau J, Dumont E, De LaRochelliere R et al. Feasibility and initial results of percutaneous aortic valve implantation including selection of the transfemoral or transapical approach in patients with severe aortic stenosis. Am J Cardiol 2008;102:1240-6.

136 Gaede L, Kim WK, Liebetrau C et al. Pacemaker implantation after TAVI: predictors of AV block persistence. Clin Res Cardiol 2018;107:60-69.

137 Tarantini G, Nai Fovino L, Tellaroli P et al. TAVR with mechanically expandable prostheses: Is balloon aortic valvuloplasty really necessary? Int J Cardiol 2017;246:37-40.

138 Mollmann H, Linke A, Holzhey DM et al. Implantation and 30-Day Follow-Up on All 4 Valve Sizes Within the Portico Transcatheter Aortic Bioprosthetic Family. JACC Cardiovasc Interv 2017;10:1538-1547.

139 Bajrangee A, Coughlan JJ, Teehan S et al. Early and mid-term outcomes after transcatheter aortic valve implantation (TAVI) in Ireland. Int J Cardiol Heart Vasc 2017;16:1-3.

140 Seeger J, Gonska B, Rottbauer W, Wohrle J. New generation devices for transfemoral transcatheter aortic valve replacement are superior compared with last generation devices with respect to VARC-2 outcome. Cardiovasc Interv Ther 2017.

141 Gerckens U, Tamburino C, Bleiziffer S et al. Final 5-year clinical and echocardiographic results for treatment of severe aortic stenosis with a self-expanding bioprosthesis from the ADVANCE Study. Eur Heart J 2017;38:2729-2738.

142 Yashima F, Yamamoto M, Tanaka M et al. Transcatheter aortic valve implantation in patients with an extremely small native aortic annulus: The OCEAN-TAVI registry. Int J Cardiol 2017;240:126-131.

143 Seeger J, Gonska B, Rottbauer W, Wohrle J. Outcome With the Repositionable and Retrievable Boston Scientific Lotus Valve Compared With the Balloon-Expandable Edwards Sapien 3 Valve in Patients Undergoing Transfemoral Aortic Valve Replacement. Circ Cardiovasc Interv 2017;10.

144 Meredith IT, Dumonteil N, Blackman DJ et al. Repositionable percutaneous aortic valve implantation with the LOTUS valve: 30-day and 1-year outcomes in 250 high-risk surgical patients. EuroIntervention 2017;13:788-795.

145 Dumonteil N, Meredith IT, Blackman DJ et al. Insights into the need for permanent pacemaker following implantation of the repositionable LOTUS valve for transcatheter aortic valve replacement in 250 patients: results from the REPRISE II trial with extended cohort. EuroIntervention 2017;13:796-803.

146 Liao YB, Zhao ZG, Wei X et al. Transcatheter aortic valve implantation with the self-expandable venus A-Valve and CoreValve devices: Preliminary Experiences in China. Catheter Cardiovasc Interv 2017;89:528-533.

147 Gonska B, Seeger J, Kessler M, von Keil A, Rottbauer W, Wohrle J. Predictors for permanent pacemaker implantation in patients undergoing transfemoral aortic valve implantation with the Edwards Sapien 3 valve. Clin Res Cardiol 2017;106:590-597.

148 Martin GP, Sperrin M, Bagur R et al. Pre-Implantation Balloon Aortic Valvuloplasty and Clinical Outcomes Following Transcatheter Aortic Valve Implantation: A Propensity Score Analysis of the UK Registry. J Am Heart Assoc 2017;6.

149 Maeno Y, Abramowitz Y, Kazuno Y et al. Transcatheter Aortic Valve Implantation With Different Valve Designs for Severe Device Landing Zone Calcification. Int Heart J 2017;58:56-62.

150 Jatene T, Castro-Filho A, Meneguz-Moreno RA et al. Prospective comparison between three TAVR devices: ACURATE neo vs. CoreValve vs. SAPIEN XT. A single heart team experience in patients with severe aortic stenosis. Catheter Cardiovasc Interv 2017;90:139-146.

151 Nara Y, Watanabe Y, Kozuma K et al. Incidence, Predictors, and Mid-Term Outcomes of Percutaneous Closure Failure After Transfemoral Aortic Valve Implantation Using an Expandable Sheath (from the Optimized Transcatheter Valvular Intervention [OCEAN-TAVI] Registry). Am J Cardiol 2017;119:611-617.

152 Naber CK, Pyxaras SA, Ince H et al. A multicentre European registry to evaluate the Direct Flow Medical transcatheter aortic valve system for the treatment of patients with severe aortic stenosis. EuroIntervention 2016;12:e1413-e1419.

153 D'Ancona G, Agma HU, Kische S et al. Introducing transcatheter aortic valve implantation with a new generation prosthesis: Institutional learning curve and effects on acute outcomes. Neth Heart J 2017;25:106-115.

154 Takimoto S, Saito N, Minakata K et al. Favorable Clinical Outcomes of Transcatheter Aortic Valve Implantation in Japanese Patients- First Report From the Post-Approval K-TAVI Registry. Circ J 2016;81:103-109.

155 Zaman S, McCormick L, Gooley R et al. Incidence and predictors of permanent pacemaker implantation following treatment with the repositionable Lotus transcatheter aortic valve. Catheter Cardiovasc Interv 2017;90:147-154.

156 Pilgrim T, Stortecky S, Nietlispach F et al. Repositionable Versus Balloon-Expandable Devices for Transcatheter Aortic Valve Implantation in Patients With Aortic Stenosis. J Am Heart Assoc 2016;5.

157 Fanning JP, Wesley AJ, Walters DL et al. Neurological Injury in Intermediate-Risk Transcatheter Aortic Valve Implantation. J Am Heart Assoc 2016;5.

158 Mauri V, Reimann A, Stern D et al. Predictors of Permanent Pacemaker Implantation After Transcatheter Aortic Valve Replacement With the SAPIEN 3. JACC Cardiovasc Interv 2016;9:2200-2209.

159 Attizzani GF, Ohno Y, Latib A et al. Acute and long-term (2-years) clinical outcomes of the CoreValve 31mm in large aortic annuli: A multicenter study. Int J Cardiol 2017;227:543-549.

160 Kische S, D'Ancona G, Agma HU et al. Trans-catheter aortic valve implantation with the direct flow medical prosthesis: Single center short-term clinical and echocardiographic outcomes. Catheter Cardiovasc Interv 2017;89:420-428.

161 Bushnaq H, Metz D, Petrov A et al. Direct aortic access for transcatheter aortic valve replacement with a fully repositionable and retrievable nonmetallic valve system. J Thorac Cardiovasc Surg 2016;152:1611-1615.

162 Puri R, Byrne J, Muller R et al. Transcatheter aortic valve implantation in patients with small aortic annuli using a 20 mm balloon-expanding valve. Heart 2017;103:148-153.

163 Kische S, D'Ancona G, Agma HU et al. Transcatheter aortic valve implantation in obese patients: Overcoming technical challenges and maintaining adequate hemodynamic performance using new generation prostheses. Int J Cardiol 2016;220:909-13.

164 Popma JJ, Gleason TG, Yakubov SJ et al. Relationship of Annular Sizing Using Multidetector Computed Tomographic Imaging and Clinical Outcomes After Self-Expanding CoreValve Transcatheter Aortic Valve Replacement. Circ Cardiovasc Interv 2016;9.

165 Watanabe Y, Kozuma K, Hioki H et al. Comparison of Results of Transcatheter Aortic Valve Implantation in Patients With Versus Without Active Cancer. Am J Cardiol 2016;118:572-7.

166 Silaschi M, Treede H, Rastan AJ et al. The JUPITER registry: 1-year results of transapical aortic valve implantation using a second-generation transcatheter heart valve in patients with aortic stenosis. Eur J Cardiothorac Surg 2016;50:874-881.

167 Wohrle J, Gonska B, Rodewald C, Seeger J, Scharnbeck D, Rottbauer W. Transfemoral Aortic Valve Implantation with the New Edwards Sapien 3 Valve for Treatment of Severe Aortic Stenosis-Impact of Valve Size in a Single Center Experience. PLoS One 2016;11:e0151247.

168 Kiramijyan S, Magalhaes MA, Koifman E et al. Aortic Regurgitation in Patients Undergoing Transcatheter Aortic Valve Replacement With the Self-Expanding CoreValve Versus the Balloon-Expandable SAPIEN XT Valve. Am J Cardiol 2016;117:1502-10.

169 Brecker SJ, Bleiziffer S, Bosmans J et al. Impact of Anesthesia Type on Outcomes of Transcatheter Aortic Valve Implantation (from the Multicenter ADVANCE Study). Am J Cardiol 2016;117:1332-8.

170 Meredith IT, Walters DL, Dumonteil N et al. 1-Year Outcomes With the Fully Repositionable and Retrievable Lotus Transcatheter Aortic Replacement Valve in 120 High-Risk Surgical Patients With Severe Aortic Stenosis: Results of the REPRISE II Study. JACC Cardiovasc Interv 2016;9:376-384.

171 Webb JG, Doshi D, Mack MJ et al. A Randomized Evaluation of the SAPIEN XT Transcatheter Heart Valve System in Patients With Aortic Stenosis Who Are Not Candidates for Surgery. JACC Cardiovasc Interv 2015;8:1797-806.

172 Iacovelli F, Pignatelli A, Giugliano G et al. Prosthesis depth and conduction disturbances after last generation balloon-expandable transcatheter aortic valve implantation. Europace 2018;20:116-123.

173 Arai T, Lefevre T, Hovasse T et al. Evaluation of the learning curve for transcatheter aortic valve implantation via the transfemoral approach. Int J Cardiol 2016;203:491-7.

174 Husser O, Kessler T, Burgdorf C et al. Conduction Abnormalities and Pacemaker Implantations After SAPIEN 3 Vs SAPIEN XT Prosthesis Aortic Valve Implantation. Rev Esp Cardiol (Engl Ed) 2016;69:141-8.

175 Krishnaswamy A, Latib A, Malik A et al. Resource utilization for transfemoral transcatheter aortic valve replacement: An international comparison. Catheter Cardiovasc Interv 2016;87:145-51.

176 Inohara T, Hayashida K, Watanabe Y et al. Streamlining the learning process for TAVI: Insight from a comparative analysis of the OCEAN-TAVI and the massy registries. Catheter Cardiovasc Interv 2016;87:963-70.

177 Subban V, Murdoch D, Savage ML et al. Outcomes of transcatheter aortic valve implantation in high surgical risk and inoperable patients with aortic stenosis: a single Australian Centre experience. Intern Med J 2016;46:42-51.

178 Fateh-Moghadam S, Voesch S, Htun P et al. Platelet activation is less enhanced in the new balloon expandable Edwards Sapien 3 valve compared to its predecessor model (Edwards Sapien XT). Thromb Haemost 2016;115:109-16.

179 Wendt D, Al-Rashid F, Kahlert P et al. Low Incidence of Paravalvular Leakage With the Balloon-Expandable Sapien 3 Transcatheter Heart Valve. Ann Thorac Surg 2015;100:819-25;discussion 825-6.

180 Petronio AS, Sinning JM, Van Mieghem N et al. Optimal Implantation Depth and Adherence to Guidelines on Permanent Pacing to Improve the Results of Transcatheter Aortic Valve Replacement With the Medtronic CoreValve System: The CoreValve Prospective, International, Post-Market ADVANCE-II Study. JACC Cardiovasc Interv 2015;8:837-846.

181 Weber M, Bruggemann E, Schueler R et al. Impact of left ventricular conduction defect with or without need for permanent right ventricular pacing on functional and clinical recovery after TAVR. Clin Res Cardiol 2015;104:964-74.

182 Collas VM, Dubois C, Legrand V et al. Midterm clinical outcome following Edwards SAPIEN or Medtronic Corevalve transcatheter aortic valve implantation (TAVI): Results of the Belgian TAVI registry. Catheter Cardiovasc Interv 2015;86:528-35.

183 Watanabe Y, Lefevre T, Bouvier E et al. Prognostic value of aortic root calcification volume on clinical outcomes after transcatheter balloon-expandable aortic valve implantation. Catheter Cardiovasc Interv 2015;86:1105-13.

184 Blackstone EH, Suri RM, Rajeswaran J et al. Propensity-matched comparisons of clinical outcomes after transapical or transfemoral transcatheter aortic valve replacement: a placement of aortic transcatheter valves (PARTNER)-I trial substudy. Circulation 2015;131:1989-2000.

185 Chang HH, Chen IM, Chen PL, Hsu TL, Lin SM, Chen YH. Comparison of balloon-expandable valves versus self-expandable valves in high-risk patients undergoing transcatheter aortic valve replacement for severe aortic stenosis. J Chin Med Assoc 2015;78:331-8.

186 Adamo M, Fiorina C, Curello S et al. Role of different vascular approaches on transcatheter aortic valve implantation outcome: a single-center study. J Cardiovasc Med (Hagerstown) 2015;16:279-85.

187 Bruschi G, De Marco F, Botta L et al. Right anterior mini-thoracotomy direct aortic self-expanding trans-catheter aortic valve implantation: A single center experience. Int J Cardiol 2015;181:437-42.

188 Ribera A, Slof J, Andrea R et al. Transfemoral transcatheter aortic valve replacement compared with surgical replacement in patients with severe aortic stenosis and comparable risk: cost-utility and its determinants. Int J Cardiol 2015;182:321-8.

189 Chevalier F, Poulin F, Lamarche Y et al. Excellent outcomes for transcatheter aortic valve replacement within 1 year of opening a low-volume centre and consideration of requirements. Can J Cardiol 2014;30:1576-82.

190 Schymik G, Tzamalis P, Bramlage P et al. Clinical impact of a new left bundle branch block following TAVI implantation: 1-year results of the TAVIK cohort. Clin Res Cardiol 2015;104:351-62.

191 D'Andrea A, Padalino R, Cocchia R et al. Effects of transcatheter aortic valve implantation on left ventricular and left atrial morphology and function. Echocardiography 2015;32:928-36.

192 Muneretto C, Bisleri G, Moggi A et al. Treating the patients in the 'grey-zone' with aortic valve disease: a comparison among conventional surgery, sutureless valves and transcatheter aortic valve replacement. Interact Cardiovasc Thorac Surg 2015;20:90-5.

193 Erdoes G, Huber C, Basciani R et al. The self-expanding Symetis Acurate does not increase cerebral microembolic load when compared to the balloon-expandable Edwards Sapien prosthesis: a transcranial Doppler study in patients undergoing transapical aortic valve implantation. PLoS One 2014;9:e108191.

194 Kasel AM, Shivaraju A, Schneider S et al. Standardized methodology for transfemoral transcatheter aortic valve replacement with the Edwards Sapien XT valve under fluoroscopy guidance. J Invasive Cardiol 2014;26:451-61.

195 Lenders GD, Collas V, Hernandez JM et al. Depth of valve implantation, conduction disturbances and pacemaker implantation with CoreValve and CoreValve Accutrak system for Transcatheter Aortic Valve Implantation, a multi-center study. Int J Cardiol 2014;176:771-5.

196 Egger F, Nurnberg M, Rohla M et al. High-degree atrioventricular block in patients with preexisting bundle branch block or bundle branch block occurring during transcatheter aortic valve implantation. Heart Rhythm 2014;11:2176-82.

197 Kasel AM, Cassese S, Ischinger T et al. A prospective, non-randomized comparison of SAPIEN XT and CoreValve implantation in two sequential cohorts of patients with severe aortic stenosis. Am J Cardiovasc Dis 2014;4:87-99.

198 Gotzmann M, Czauderna A, Hehnen T et al. Three-year outcomes after transcatheter aortic valve implantation with the CoreValve prosthesis. Am J Cardiol 2014;114:606-11.

199 Watanabe Y, Hayashida K, Takayama M et al. First direct comparison of clinical outcomes between European and Asian cohorts in transcatheter aortic valve implantation: the Massy study group vs. the PREVAIL JAPAN trial. J Cardiol 2015;65:112-6.

200 Pascual I, Avanzas P, Munoz-Garcia AJ et al. Percutaneous implantation of the CoreValve(R) self-expanding valve prosthesis in patients with severe aortic stenosis and porcelain aorta: medium-term follow-up. Rev Esp Cardiol (Engl Ed) 2013;66:775-81.

201 Fuku Y, Goto T, Komiya T et al. Thirty-day outcome of transcatheter aortic valve implantation with the edwards SAPIEN XT prosthesis via the transiliofemoral approach. Circ J 2014;78:1357-63.

202 Orvin K, Dvir D, Weiss A et al. Comprehensive prospective cognitive and physical function assessment in elderly patients undergoing transcatheter aortic valve implantation. Cardiology 2014;127:227-35.

203 Greif M, Lange P, Nabauer M et al. Transcutaneous aortic valve replacement with the Edwards SAPIEN XT and Medtronic CoreValve prosthesis under fluoroscopic guidance and local anaesthesia only. Heart 2014;100:691-5.

204 Blackman DJ, Baxter PD, Gale CP et al. Do outcomes from transcatheter aortic valve implantation vary according to access route and valve type? The UK TAVI Registry. J Interv Cardiol 2014;27:86-95.

205 Sundermann SH, Grunenfelder J, Corti R et al. Outcome of patients treated with Engager transapical aortic valve implantation: one-year results of the feasibility study. Innovations (Phila) 2013;8:332-6.

206 Schewel D, Frerker C, Schewel J et al. Clinical impact of paravalvular leaks on biomarkers and survival after transcatheter aortic valve implantation. Catheter Cardiovasc Interv 2015;85:502-14.

207 Mack MJ, Brennan JM, Brindis R et al. Outcomes following transcatheter aortic valve replacement in the United States. JAMA 2013;310:2069-77.

208 Schofer J, Colombo A, Klugmann S et al. Prospective multicenter evaluation of the direct flow medical transcatheter aortic valve. J Am Coll Cardiol 2014;63:763-8.

209 Munoz-Garcia AJ, del Valle R, Trillo-Nouche R et al. The Ibero-American transcatheter aortic valve implantation registry with the CoreValve prosthesis. Early and long-term results. Int J Cardiol 2013;169:359-65.

210 Bedogni F, Latib A, De Marco F et al. Interplay between mitral regurgitation and transcatheter aortic valve replacement with the CoreValve Revalving System: a multicenter registry. Circulation 2013;128:2145-53.

211 Papadopoulos N, Ilioska P, Fichtlscherer S et al. Transapical aortic valve implantation in patients with previous cardiac surgery. Ann Thorac Surg 2014;97:37-42.

212 Holzhey D, Linke A, Treede H et al. Intermediate follow-up results from the multicenter engager European pivotal trial. Ann Thorac Surg 2013;96:2095-100.

213 Watanabe Y, Hayashida K, Yamamoto M et al. Transfemoral aortic valve implantation in patients with an annulus dimension suitable for either the Edwards valve or the CoreValve. Am J Cardiol 2013;112:707-13.

214 Mendiz OA, Fraguas H, Lev GA, Valdivieso LR, Favaloro RR. Transcatheter aortic valve implantation without balloon predilation: a single-center pilot experience. Catheter Cardiovasc Interv 2013;82:292-7.

215 Meguro K, Lellouche N, Yamamoto M et al. Prognostic value of QRS duration after transcatheter aortic valve implantation for aortic stenosis using the CoreValve. Am J Cardiol 2013;111:1778-83.

216 Testa L, Latib A, De Marco F et al. Clinical impact of persistent left bundle-branch block after transcatheter aortic valve implantation with CoreValve Revalving System. Circulation 2013;127:1300-7.

217 Spargias K, Toutouzas K, Chrissoheris M et al. The Athens TAVR Registry of newer generation transfemoral aortic valves: 30-day outcomes. Hellenic J Cardiol 2013;54:18-24.

218 Parenica J, Nemec P, Tomandl J et al. Prognostic utility of biomarkers in predicting of one-year outcomes in patients with aortic stenosis treated with transcatheter or surgical aortic valve implantation. PLoS One 2012;7:e48851.

219 Borz B, Durand E, Godin M et al. Incidence, predictors and impact of bleeding after transcatheter aortic valve implantation using the balloon-expandable Edwards prosthesis. Heart 2013;99:860-5.

220 Munoz-Garcia AJ, Munoz-Garcia M, Carrasco-Chinchilla F et al. Incidence and clinical outcome of prosthesis-patient mismatch after transcatheter aortic valve implantation with the CoreValve prosthesis. Int J Cardiol 2013;167:1074-6.

221 Kala P, Tretina M, Poloczek M et al. Quality of life after transcatheter aortic valve implantation and surgical replacement in high-risk elderly patients. Biomed Pap Med Fac Univ Palacky Olomouc Czech Repub 2013;157:75-80.

222 Kahlert P, Al-Rashid F, Dottger P et al. Cerebral embolization during transcatheter aortic valve implantation: a transcranial Doppler study. Circulation 2012;126:1245-55.

223 Genereux P, Webb JG, Svensson LG et al. Vascular complications after transcatheter aortic valve replacement: insights from the PARTNER (Placement of AoRTic TraNscathetER Valve) trial. J Am Coll Cardiol 2012;60:1043-52.

224 Wendler O, Walther T, Schroefel H et al. Transapical aortic valve implantation: mid-term outcome from the SOURCE registry. Eur J Cardiothorac Surg 2013;43:505-11; discussion 511-2.

225 Bruschi G, de Marco F, Botta L et al. Direct aortic access for transcatheter self-expanding aortic bioprosthetic valves implantation. Ann Thorac Surg 2012;94:497-503.

226 Nielsen HH, Klaaborg KE, Nissen H et al. A prospective, randomised trial of transapical transcatheter aortic valve implantation vs. surgical aortic valve replacement in operable elderly patients with aortic stenosis: the STACCATO trial. EuroIntervention 2012;8:383-9.

227 Staubach S, Franke J, Gerckens U et al. Impact of aortic valve calcification on the outcome of transcatheter aortic valve implantation: results from the prospective multicenter German TAVI registry. Catheter Cardiovasc Interv 2013;81:348-55.

228 Durand E, Borz B, Godin M et al. Transfemoral aortic valve replacement with the Edwards SAPIEN and Edwards SAPIEN XT prosthesis using exclusively local anesthesia and fluoroscopic guidance: feasibility and 30-day outcomes. JACC Cardiovasc Interv 2012;5:461-467.

229 Gilard M, Eltchaninoff H, Iung B et al. Registry of transcatheter aortic-valve implantation in high-risk patients. N Engl J Med 2012;366:1705-15.

230 Wendler O, Thielmann M, Schroefel H et al. Worldwide experience with the 29-mm Edwards SAPIEN XT transcatheter heart valve in patients with large aortic annulus. Eur J Cardiothorac Surg 2013;43:371-7.

231 Eltchaninoff H, Durand E, Borz B et al. Prospective analysis of 30-day safety and performance of transfemoral transcatheter aortic valve implantation with Edwards SAPIEN XT versus SAPIEN prostheses. Arch Cardiovasc Dis 2012;105:132-40.

232 Modine T, Sudre A, Delhaye C et al. Transcutaneous aortic valve implantation using the left carotid access: feasibility and early clinical outcomes. Ann Thorac Surg 2012;93:1489-94.

233 Gasparetto V, Fraccaro C, Tarantini G et al. Safety and effectiveness of a selective strategy for coronary artery revascularization before transcatheter aortic valve implantation. Catheter Cardiovasc Interv 2013;81:376-83.

234 Samim M, Stella PR, Agostoni P et al. A prospective "oversizing" strategy of the Edwards SAPIEN bioprosthesis: results and impact on aortic regurgitation. J Thorac Cardiovasc Surg 2013;145:398-405.

235 Walther T, Thielmann M, Kempfert J et al. PREVAIL TRANSAPICAL: multicentre trial of transcatheter aortic valve implantation using the newly designed bioprosthesis (SAPIEN-XT) and delivery system (ASCENDRA-II). Eur J Cardiothorac Surg 2012;42:278-83; discussion 283.

236 Leber AW, Kasel M, Ischinger T et al. Aortic valve calcium score as a predictor for outcome after TAVI using the CoreValve revalving system. Int J Cardiol 2013;166:652-7.

237 Calvi V, Conti S, Pruiti GP et al. Incidence rate and predictors of permanent pacemaker implantation after transcatheter aortic valve implantation with self-expanding CoreValve prosthesis. J Interv Card Electrophysiol 2012;34:189-95.

238 Saia F, Lemos PA, Bordoni B et al. Transcatheter aortic valve implantation with a self-expanding nitinol bioprosthesis: prediction of the need for permanent pacemaker using simple baseline and procedural characteristics. Catheter Cardiovasc Interv 2012;79:712-9.

239 Guetta V, Goldenberg G, Segev A et al. Predictors and course of high-degree atrioventricular block after transcatheter aortic valve implantation using the CoreValve Revalving System. Am J Cardiol 2011;108:1600-5.

240 Kalavrouziotis D, Rodes-Cabau J, Bagur R et al. Transcatheter aortic valve implantation in patients with severe aortic stenosis and small aortic annulus. J Am Coll Cardiol 2011;58:1016-24.

241 Genereux P, Kodali S, Leon MB et al. Clinical outcomes using a new crossover balloon occlusion technique for percutaneous closure after transfemoral aortic valve implantation. JACC Cardiovasc Interv 2011;4:861-7.

242 Gotzmann M, Pljakic A, Bojara W et al. Transcatheter aortic valve implantation in patients with severe symptomatic aortic valve stenosis-predictors of mortality and poor treatment response. Am Heart J 2011;162:238-245 e1.

243 Georgiadou P, Kontodima P, Sbarouni E et al. Long-term quality of life improvement after transcatheter aortic valve implantation. Am Heart J 2011;162:232-7.

244 Gotzmann M, Lindstaedt M, Bojara W, Ewers A, Mugge A. Clinical outcome of transcatheter aortic valve implantation in patients with low-flow, low gradient aortic stenosis. Catheter Cardiovasc Interv 2012;79:693-701.

245 Takagi K, Latib A, Al-Lamee R et al. Predictors of moderate-to-severe paravalvular aortic regurgitation immediately after CoreValve implantation and the impact of postdilatation. Catheter Cardiovasc Interv 2011;78:432-43.

246 Grube E, Naber C, Abizaid A et al. Feasibility of transcatheter aortic valve implantation without balloon pre-dilation: a pilot study. JACC Cardiovasc Interv 2011;4:751-7.

247 Walther T, Kempfert J, Rastan A et al. Transapical aortic valve implantation at 3 years. J Thorac Cardiovasc Surg 2012;143:326-31.

248 Van Mieghem NM, Nuis RJ, Tzikas A et al. Prevalence and prognostic implications of baseline anaemia in patients undergoing transcatheter aortic valve implantation. EuroIntervention 2011;7:184-91.

249 Wenaweser P, Pilgrim T, Roth N et al. Clinical outcome and predictors for adverse events after transcatheter aortic valve implantation with the use of different devices and access routes. Am Heart J 2011;161:1114-24.

250 Nuis RJ, Van Mieghem NM, Schultz CJ et al. Timing and potential mechanisms of new conduction abnormalities during the implantation of the Medtronic CoreValve System in patients with aortic stenosis. Eur Heart J 2011;32:2067-74.

251 Nuis RJ, Piazza N, Van Mieghem NM et al. In-hospital complications after transcatheter aortic valve implantation revisited according to the Valve Academic Research Consortium definitions. Catheter Cardiovasc Interv 2011;78:457-67.

252 Rubin JM, Avanzas P, del Valle R et al. Atrioventricular conduction disturbance characterization in transcatheter aortic valve implantation with the CoreValve prosthesis. Circ Cardiovasc Interv 2011;4:280-6.

253 Nuis RJ, van Mieghem NM, van der Boon RM et al. Effect of experience on results of transcatheter aortic valve implantation using a Medtronic CoreValve System. Am J Cardiol 2011;107:1824-9.

254 Aktug O, Dohmen G, Brehmer K et al. Incidence and predictors of left bundle branch block after transcatheter aortic valve implantation. Int J Cardiol 2012;160:26-30.

255 Gotzmann M, Bojara W, Lindstaedt M et al. One-year results of transcatheter aortic valve implantation in severe symptomatic aortic valve stenosis. Am J Cardiol 2011;107:1687-92.

256 Abdel-Wahab M, Zahn R, Horack M et al. Aortic regurgitation after transcatheter aortic valve implantation: incidence and early outcome. Results from the German transcatheter aortic valve interventions registry. Heart 2011;97:899-906.

257 Bosmans JM, Kefer J, De Bruyne B et al. Procedural, 30-day and one year outcome following CoreValve or Edwards transcatheter aortic valve implantation: results of the Belgian national registry. Interact Cardiovasc Thorac Surg 2011;12:762-7.

258 Danenberg H, Finkelstein A, Kornowski R et al. Percutaneous implantation of the self-expandable CoreValve for high risk patients with severe aortic valve stenosis: early Israeli experience. Isr Med Assoc J 2010;12:468-71.

259 Fraccaro C, Buja G, Tarantini G et al. Incidence, predictors, and outcome of conduction disorders after transcatheter self-expandable aortic valve implantation. Am J Cardiol 2011;107:747-54.

260 Lopez-Otero D, Munoz-Garcia AJ, Avanzas P et al. Axillary approach for transcatheter aortic valve implantation: optimization of the endovascular treatment for the aortic valve stenosis. Rev Esp Cardiol 2011;64:121-6.

261 Hernandez-Antolin RA, Garcia E, Sandoval S et al. Findings of a mixed transfemoral aortic valve implantation program using Edwards and CoreValve devices. Rev Esp Cardiol 2011;64:35-42.

262 Wendler O, Walther T, Schroefel H et al. The SOURCE Registry: what is the learning curve in trans-apical aortic valve implantation? Eur J Cardiothorac Surg 2011;39:853-9; discussion 859-60.

263 Sinning JM, Ghanem A, Steinhauser H et al. Renal function as predictor of mortality in patients after percutaneous transcatheter aortic valve implantation. JACC Cardiovasc Interv 2010;3:1141-9.

264 Lefevre T, Kappetein AP, Wolner E et al. One year follow-up of the multi-centre European PARTNER transcatheter heart valve study. Eur Heart J 2011;32:148-57.

265 Nuis RJ, Van Mieghem NM, Tzikas A et al. Frequency, determinants, and prognostic effects of acute kidney injury and red blood cell transfusion in patients undergoing transcatheter aortic valve implantation. Catheter Cardiovasc Interv 2011;77:881-9.

266 Roten L, Wenaweser P, Delacretaz E et al. Incidence and predictors of atrioventricular conduction impairment after transcatheter aortic valve implantation. Am J Cardiol 2010;106:1473-80.

267 Chodor P, Wilczek K, Przybylski R et al. Immediate and 6-month outcomes of transapical and transfemoral Edwards-Sapien prosthesis implantation in patients with aortic stenosis. Kardiol Pol 2010;68:1124-31.

268 Yucel G, Paker T, Akcevin A et al. [Transcatheter aortic valve implantation: the first applications and early results in Turkey]. Turk Kardiyol Dern Ars 2010;38:258-63.

269 Haworth P, Behan M, Khawaja M et al. Predictors for permanent pacing after transcatheter aortic valve implantation. Catheter Cardiovasc Interv 2010;76:751-6.

270 Zahn R, Gerckens U, Grube E et al. Transcatheter aortic valve implantation: first results from a multi-centre real-world registry. Eur Heart J 2011;32:198-204.

271 Eltchaninoff H, Prat A, Gilard M et al. Transcatheter aortic valve implantation: early results of the FRANCE (FRench Aortic National CoreValve and Edwards) registry. Eur Heart J 2011;32:191-7.

272 Godin M, Eltchaninoff H, Furuta A et al. Frequency of conduction disturbances after transcatheter implantation of an Edwards Sapien aortic valve prosthesis. Am J Cardiol 2010;106:707-12.

273 Dworakowski R, MacCarthy PA, Monaghan M et al. Transcatheter aortic valve implantation for severe aortic stenosis-a new paradigm for multidisciplinary intervention: a prospective cohort study. Am Heart J 2010;160:237-43.

274 Petronio AS, De Carlo M, Bedogni F et al. Safety and efficacy of the subclavian approach for transcatheter aortic valve implantation with the CoreValve revalving system. Circ Cardiovasc Interv 2010;3:359-66.

275 Thomas M, Schymik G, Walther T et al. Thirty-day results of the SAPIEN aortic Bioprosthesis European Outcome (SOURCE) Registry: A European registry of transcatheter aortic valve implantation using the Edwards SAPIEN valve. Circulation 2010;122:62-9.

276 Treede H, Tubler T, Reichenspurner H et al. Six-month results of a repositionable and retrievable pericardial valve for transcatheter aortic valve replacement: the Direct Flow Medical aortic valve. J Thorac Cardiovasc Surg 2010;140:897-903.

277 Bauer F, Lemercier M, Zajarias A, Tron C, Eltchaninoff H, Cribier A. Immediate and long-term echocardiographic findings after transcatheter aortic valve implantation for the treatment of aortic stenosis: the Cribier-Edwards/Edwards-Sapien valve experience. J Am Soc Echocardiogr 2010;23:370-6.

278 John D, Buellesfeld L, Yuecel S et al. Correlation of Device landing zone calcification and acute procedural success in patients undergoing transcatheter aortic valve implantations with the self-expanding CoreValve prosthesis. JACC Cardiovasc Interv 2010;3:233-43.

279 Avanzas P, Munoz-Garcia AJ, Segura J et al. Percutaneous implantation of the CoreValve self-expanding aortic valve prosthesis in patients with severe aortic stenosis: early experience in Spain. Rev Esp Cardiol 2010;63:141-8.

280 Buellesfeld L, Wenaweser P, Gerckens U et al. Transcatheter aortic valve implantation: predictors of procedural success--the Siegburg-Bern experience. Eur Heart J 2010;31:984-91.

281 Grube E, Buellesfeld L, Mueller R et al. Progress and current status of percutaneous aortic valve replacement: results of three device generations of the CoreValve Revalving system. Circ Cardiovasc Interv 2008;1:167-75.

282 Gotzmann M, Hehen T, Germing A et al. Short-term effects of transcatheter aortic valve implantation on neurohormonal activation, quality of life and 6-minute walk test in severe and symptomatic aortic stenosis. Heart 2010;96:1102-6.

283 Gutierrez M, Rodes-Cabau J, Bagur R et al. Electrocardiographic changes and clinical outcomes after transapical aortic valve implantation. Am Heart J 2009;158:302-8.

284 Al-Attar N, Ghodbane W, Himbert D et al. Unexpected complications of transapical aortic valve implantation. Ann Thorac Surg 2009;88:90-4.

285 Ussia GP, Mule M, Barbanti M et al. Quality of life assessment after percutaneous aortic valve implantation. Eur Heart J 2009;30:1790-6.

286 Jilaihawi H, Chin D, Vasa-Nicotera M et al. Predictors for permanent pacemaker requirement after transcatheter aortic valve implantation with the CoreValve bioprosthesis. Am Heart J 2009;157:860-6.

287 Spargias K, Manginas A, Pavlides G et al. Transcatheter aortic valve implantation: first Greek experience. Hellenic J Cardiol 2008;49:397-407.

288 Behan M, Haworth P, Hutchinson N, Trivedi U, Laborde JC, Hildick-Smith D. Percutaneous aortic valve implants under sedation: our initial experience. Catheter Cardiovasc Interv 2008;72:1012-5.

289 Schofer J, Schluter M, Treede H et al. Retrograde transarterial implantation of a nonmetallic aortic valve prosthesis in high-surgical-risk patients with severe aortic stenosis: a first-in-man feasibility and safety study. Circ Cardiovasc Interv 2008;1:126-33.

290 Berry C, Oukerraj L, Asgar A et al. Role of transesophageal echocardiography in percutaneous aortic valve replacement with the CoreValve Revalving system. Echocardiography 2008;25:840-8.

291 Walther T, Falk V, Kempfert J et al. Transapical minimally invasive aortic valve implantation; the initial 50 patients. Eur J Cardiothorac Surg 2008;33:983-8.

292 Grube E, Laborde JC, Gerckens U et al. Percutaneous implantation of the CoreValve self-expanding valve prosthesis in high-risk patients with aortic valve disease: the Siegburg first-in-man study. Circulation 2006;114:1616-24.

293 Garg A, Parashar A, Agarwal S et al. Comparison of acute elastic recoil between the SAPIEN-XT and SAPIEN valves in transfemoral-transcatheter aortic valve replacement. Catheter Cardiovasc Interv 2015;85:490-6.

294 Blanke P, Pibarot P, Hahn R et al. Computed Tomography-Based Oversizing Degrees and Incidence of Paravalvular Regurgitation of a New Generation Transcatheter Heart Valve. JACC Cardiovasc Interv 2017;10:810-820.

295 Herrmann HC, Thourani VH, Kodali SK et al. One-Year Clinical Outcomes With SAPIEN 3 Transcatheter Aortic Valve Replacement in High-Risk and Inoperable Patients With Severe Aortic Stenosis. Circulation 2016;134:130-40.

296 Avanzas P, Pascual I, Munoz-Garcia AJ et al. Long-term Follow-up of Patients With Severe Aortic Stenosis Treated With a Self-expanding Prosthesis. Rev Esp Cardiol (Engl Ed) 2017;70:247-253.

297 Ngo A, Hassager C, Thyregod HGH et al. Differences in left ventricular remodelling in patients with aortic stenosis treated with transcatheter aortic valve replacement with corevalve prostheses compared to surgery with porcine or bovine biological prostheses. Eur Heart J Cardiovasc Imaging 2018;19:39-46.

298 Deeb GM, Reardon MJ, Chetcuti S et al. 3-Year Outcomes in High-Risk Patients Who Underwent Surgical or Transcatheter Aortic Valve Replacement. J Am Coll Cardiol 2016;67:2565-74.

299 Yakubov SJ, Adams DH, Watson DR et al. 2-Year Outcomes After Iliofemoral Self-Expanding Transcatheter Aortic Valve Replacement in Patients With Severe Aortic Stenosis Deemed Extreme Risk for Surgery. J Am Coll Cardiol 2015;66:1327-34.

300 Bijuklic K, Tuebler T, Reichenspurner H et al. Midterm stability and hemodynamic performance of a transfemorally implantable nonmetallic, retrievable, and repositionable aortic valve in patients with severe aortic stenosis. Up to 2-year follow-up of the direct-flow medical valve: a pilot study. Circ Cardiovasc Interv 2011;4:595-601.

301 Jilaihawi H, Chin D, Spyt T et al. Comparison of complete versus incomplete stent frame expansion after transcatheter aortic valve implantation with Medtronic CoreValve bioprosthesis. Am J Cardiol 2011;107:1830-7.
